# A genome-wide CRISPR screening uncovers that TOB1 acts as a key host factor for FMDV infection via both IFN and EGFR mediated pathways

**DOI:** 10.1371/journal.ppat.1012104

**Published:** 2024-03-21

**Authors:** Gaochuang Peng, Tianran Liu, Xiaolan Qi, Yuzhe Wang, Jingjing Ren, Jiangling Peng, Xuguang Du, Siyu Hu, Sen Wu, Yaofeng Zhao, Dan Li, Haixue Zheng

**Affiliations:** 1 State Key Laboratory of Animal Biotech Breeding, College of Biological Sciences, National Engineering Laboratory for Animal Breeding, Frontiers Science Center for Molecular Design Breeding, China Agricultural University, Beijing, China; 2 State Key Laboratory for Animal Disease Control and Prevention, College of Veterinary Medicine, Lanzhou University, Lanzhou Veterinary Research Institute, Chinese Academy of Agricultural Sciences, Lanzhou, China; University of California, Irvine, UNITED STATES

## Abstract

The interaction between foot-and-mouth disease virus (FMDV) and the host is extremely important for virus infection, but there are few researches on it, which is not conducive to vaccine development and FMD control. In this study, we designed a porcine genome-scale CRISPR/Cas9 knockout library containing 93,859 single guide RNAs targeting 16,886 protein-coding genes, 25 long ncRNAs, and 463 microRNAs. Using this library, several previously unreported genes required for FMDV infection are highly enriched post-FMDV selection in IBRS-2 cells. Follow-up studies confirmed the dependency of FMDV on these genes, and we identified a functional role for one of the FMDV-related host genes: *TOB1* (Transducer of ERBB2.1). *TOB1*-knockout significantly inhibits FMDV infection by positively regulating the expression of RIG-I and MDA5. We further found that *TOB1*-knockout led to more accumulation of mRNA transcripts of transcription factor CEBPA, and thus its protein, which further enhanced transcription of *RIG-I* and *MDA5* genes. In addition, *TOB1*-knockout was shown to inhibit FMDV adsorption and internalization mediated by EGFR/ERBB2 pathway. Finally, the FMDV lethal challenge on *TOB1*-knockout mice confirmed that the deletion of *TOB1* inhibited FMDV infection *in vivo*. These results identify TOB1 as a key host factor involved in FMDV infection in pigs.

## Author summary

Host factors play a critical role in FMDV infection, however, the mechanisms are poorly understood. Aiming to comprehensively study the host factors involved in FMDV infection, we develop a porcine genome-scale CRISPR/Cas9 knockout library and successfully confirmed that the knockout of *TOB1* (Transducer of ERBB2.1) could significantly inhibit FMDV infection. On one hand, *TOB1*-knockout could promote the expression of RIG-I and MDA5, and thus IFNB and ISGs (interferon stimulating genes), thereby inhibiting FMDV infection. On the other hand, *TOB1*-knockout inhibits FMDV entry mediated by EGFR/ERBB2 pathway. These findings reveal TOB1 as a key host factor mediating FMDV infection in pigs.

## Introduction

Foot-and-mouth disease (FMD) is highly contagious and severe disease affecting pigs, cattle, sheep, and other cloven-hoofed animals [1]. The disease is characterized by blisters in the mouth, nose, and hooves, accompanied by depression, salivation, limping, and sometimes even death in young livestock [2–4]. Due to its high infectivity and multiple transmission routes, FMD has caused significant economic losses and social impact [5], and is listed as one of the legally reported animal infectious diseases by the World Organization for Animal Health (WOAH, founded as OIE).

The pathogen of FMD is foot-and-mouth disease virus (FMDV), a single-stranded positive-strand RNA virus that infects a broad range of hosts and has a high genetic variability. The FMDV infection cycle involves binding to integrin and heparan sulfate [6–9], followed by viral entry, replication, and assembly, ultimately leading to the release of viral particles from infected cells [10]. Recent studies have made progress in identifying viral components required for FMDV infection [11–13], but less is known about the host proteins involved. FMDV has evolved various strategies to escape host immune responses, and host restriction factors can interfere with different stages of the viral life cycle to inhibit infection [14]. For example, the host gene early growth response gene-1 (EGR1) promotes the production of type I interferon (IFN-I) by increasing the phosphorylation of TBK1 and inhibits FMDV replication [15], while DnaJ heat shock protein family member A3 (DNAJA3) degrades FMDV VP1 through lysosomal pathway and increases IFN-I response to inhibit FMDV replication [16]. Studying the interaction between FMDV and its host can help identify potential antiviral targets and aid in the development of efficient vaccines and disease-resistant breeding technologies.

With the development of gene editing technology, genome-wide screening of CRISPR has been widely used in the study of host factors associated with viral infection [17,18]. In recent years, CRISPR-based screening strategies have successfully identified many key host genes, particularly genes encoding receptors required for DENV (Dengue virus), ZIKV (Zika virus), WNV (West Nile virus), HIV, and HCV (Hepatitis C virus) [19–22]. Therefore, this study utilized a genome-wide CRISPR knockout screen system to identify host genes related to FMDV infection and analyze the regulatory network between FMDV and the host in-depth.

In this study, we designed a porcine genome-scale CRISPR/Cas9 knockout library containing 93,859 single guide RNAs targeting 16,886 protein-coding genes, 25 long ncRNAs, and 463 microRNAs. We performed a positive selection screening by exposing the mutant cell library to FMDV challenge and successfully confirmed that the knockout of *TOB1* (Transducer of ERBB2.1) could significantly inhibit FMDV infection. We further found that *TOB1*-knockout inhibited FMDV infection in various pig cell lines by positively regulating RIG-I and MDA5-mediated innate immune responses and inhibiting EGFR/ERBB2-mediated viral entry.

## Results

### A high throughput CRISPR screen for FMDV resistance genes

**Fig 1:**
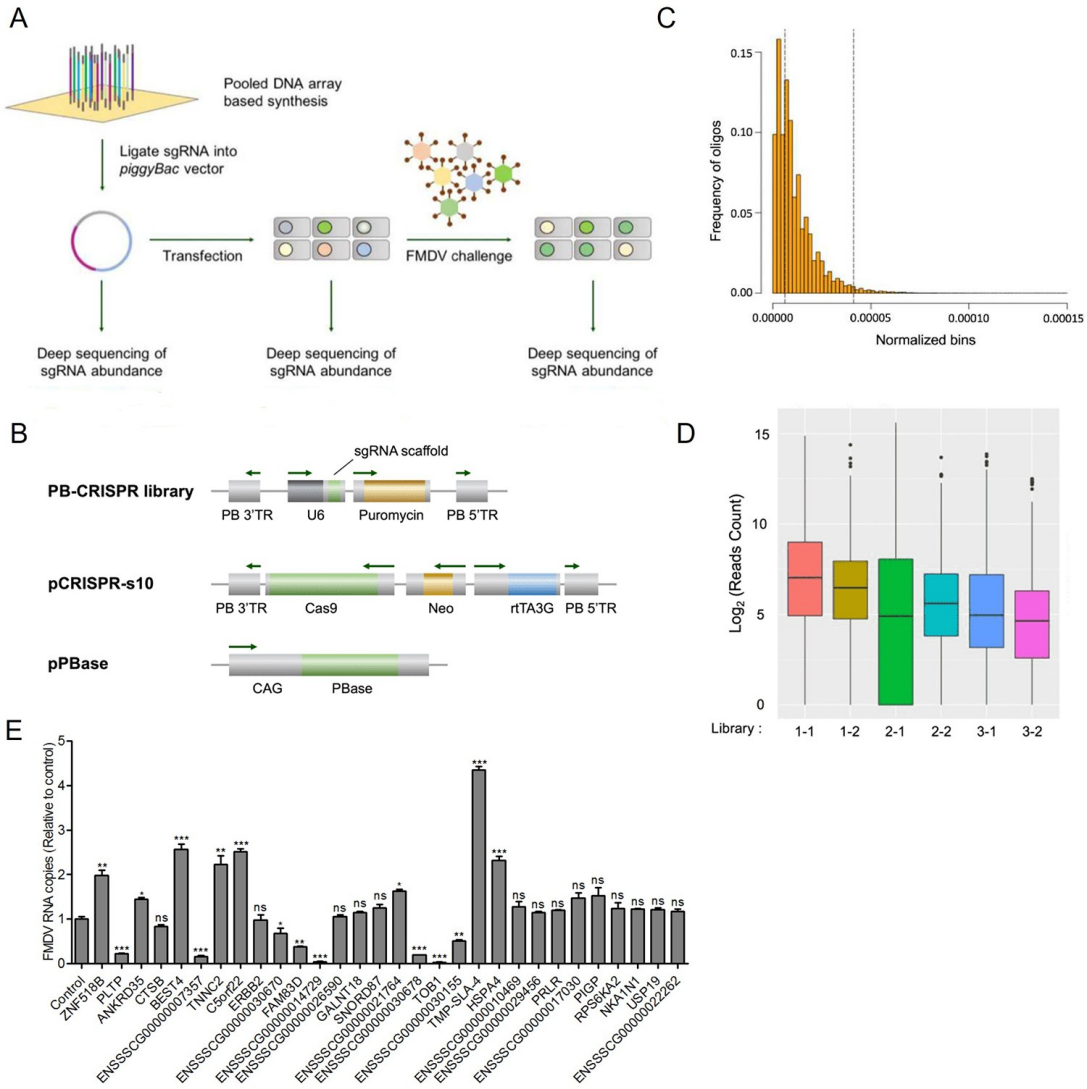
Strategy for identifying essential genes for FMDV infection. **A** The process of CRISPR screening for host genes associated with FMDV infection. These designed sgRNA constructs were synthesized as sgRNA oligos, which were subsequently cloned into *piggyBac* vectors. IBRS-2 cells were transfected with library plasmids to obtain the mutant cell library, subsequently exposing to FMDV infection. **B** Library plasmids including PB-CRISPR library, *piggyBac* transposase expression vector pPBase, and doxycycline-induced Cas9 expression vector pCRISPR-S10. **C** Sequencing result of sgRNAs targeting sequences in plasmid pools libraries. **D** The post-FMDV infection mutant cell populations containing the whole CRISPR pooled sgRNA library were characterized using next-generation sequencing. (1–1, 1–2, 1–3) and (2–1, 2–2, 2–3) were two independent FMDV infection experiments, with three biological replicates in each group. **E** Thirty of the candidate gene-knockout monoclonal cell lines were infected with FMDV at 0.1 MOI for 12 h. The FMDV RNA copies were measured by absolute quantitative real-time PCR. Control, the wild-type cells transfected with non-targeting sgRNA. Data shown includes technical replicates from a single experiment and is representative of three independent experiments (E). Data are represented as means ± S.D.; *P < 0.05; **P < 0.01; ***P < 0.001; ns, no significant. *P* values were determined by two-sided Student’s *t*-test.

To target the entire porcine genome, we designed a PB-CRISPR/Cas9 knockout library containing 93,859 sgRNAs targeting 16,886 protein-coding genes. The construction and screening process of PB-CRISPR/Cas9 knockout library is shown in Fig 1A. The sgRNA sequences were synthesized as an oligo array and subsequently cloned into PB-CRISPR-sg4 vectors using Gibson assembly (Fig 1B). To verify the quality and coverage of PB-CRISPR library, we subjected the library to next-generation sequencing (NGS) after PCR amplification. We detected 89,150 unique sgRNAs, accounting for 94.98% of the total designed sgRNAs. The sgRNAs in the PB-CRISPR library were uniformly distributed and of good quality (Fig 1C), making it suitable for the construction of a genome-wide mutant cell library. Then we used the *piggyBac* transposon-mediated two-plasmid system (PB-CRISPR and pCRISPR-S10) [23] to construct a genome-wide mutant cell library in the IBRS-2 cell line (Fig 1B).

We transfected PB-CRISPR, pCRISPR-S10, and *piggyBac* transposase expression plasmids into IBRS-2 cells to obtain a genome-wide mutant cell library (S1B Fig). After resistance selection and doxycycline induction, the expression of Cas9 mRNA and protein in the library cells treated with doxycycline was higher than in the control group (S1A Fig). The coverage ratio of the unique sgRNAs detected in the mutant cell library to the total designed sgRNAs was approximately 85% (Table 1). We performed positive screening by exposing the mutant cell library to FMDV infection and retaining the FMDV-resistant cells (Figs 1A and S1C). PCR amplification and deep sequencing detected enrichment of certain sgRNAs representing their respective targeted genes in the FMDV-resistant cells. The genomic DNA of FMDV-resistant cells was isolated and used as the template for PCR amplification, and the abundance of sgRNAs in each group of NGS results was counted. It was found that sgRNAs were significantly enriched in the FMDV-resistant cells (Fig 1D). As each sgRNA correspond to a specific host gene, we identified and ranked candidate genes based on the abundance of sgRNAs, and gene scores were calculated. The top 30 candidate genes were sorted according to gene scores, and their information was shown in Table 2.

**Table 1:** The number of unique sgRNAs in mutant cell library.

Cell Library	Unique sgRNA number	Unique sgRNA/total sgRNA	Maximum reads of unique sgRNA
Library 1	80,005	85.24%	367
Library 2	80,892	86.18%	442
Library 3	78,100	83.21%	355

To obtain the mutant cell library, the PB-CRISPR library, pCRISPR-S10 and piggyBac transposase expression plasmids were co-transfected into IBRS-2 cells. Library 1, Library 2, and Library 3 were three independent biological replicates.

**Table 2:** Score and ranking of top 30 candidate genes.

Rank	Gene name	Ensemble version	Score
1	ZNF518B	ENSSSCG00000029711	7.530178
2	PLTP	ENSSSCG00000007435	6.000000
3	ANKRD35	ENSSSCG00000006688	5.679854
4	CTSB	ENSSSCG00000023666	5.590730
5	BEST4	ENSSSCG00000003929	5.435334
6	ENSSSCG00000007357	ENSSSCG00000007357	5.376751
7	TNNC2	ENSSSCG00000007424	5.288235
8	C5orf22	ENSSSCG00000016808	5.036212
9	ErbB2	ENSSSCG00000017497	5.028724
10	ENSSSCG00000030670	ENSSSCG00000030670	4.991400
11	FAM83D	ENSSSCG00000007351	4.860121
12	ENSSSCG00000014729	ENSSSCG00000014729	4.808549
13	ENSSSCG00000026590	ENSSSCG00000026590	4.739929
14	GALNT18	ENSSSCG00000013403	4.738975
15	SNORD87	ENSSSCG00000018868	4.703335
16	ENSSSCG00000021764	ENSSSCG00000021764	4.687823
17	ENSSSCG00000030678	ENSSSCG00000030678	4.651306
18	TOB1	ENSSSCG00000017558	4.647817
19	ENSSSCG00000030155	ENSSSCG00000030155	4.617623
20	TMP-SLA-4	ENSSSCG00000030873	4.571541
21	HSPA4	ENSSSCG00000014292	4.567191
22	ENSSSCG00000010469	ENSSSCG00000010469	4.559091
23	ENSSSCG00000029456	ENSSSCG00000029456	4.551603
24	PRLR	ENSSSCG00000016830	4.544850
25	ENSSSCG00000017030	ENSSSCG00000017030	4.515415
26	PIGP	ENSSSCG00000012061	4.484656
27	RPS6KA2	ENSSSCG00000004022	4.464200
28	NKAIN1	ENSSSCG00000003593	4.453457
29	USP19	ENSSSCG00000011375	4.434152
30	ENSSSCG00000022262	ENSSSCG00000022262	4.386158

The integrated sgRNAs information based on NGS results of cells with FMDV resistance was analyzed, and corresponding genes were ranked based on the abundance of sgRNAs. Subsequently, gene scores were calculated based on the ranking of sgRNAs, the number of repetitions of the same sgRNA in different experimental groups, as well as the number and ranking of different sgRNAs targeting the same gene. It is worth noting that in the above ranking table, we have removed some non-protein-coding genes.

Practically, the whole genome CRISPR screening may generate false positive results. To confirm the association of the candidate genes with FMDV infection, we thus generated gene-knockout cell lines for thirty of the candidate genes (S1D Fig). Among the 30 genes, we successfully confirmed that the knockout of seven genes including *TOB1*, *PLTP*, etc., could significantly inhibit FMDV infection in IBRS-2 cells (Fig 1E), which suggested that TOB1 play an important role in FMDV infection.

### *TOB1*-knockout significantly inhibits FMDV infection in three cell lines

TOB1 is a tumor-suppressing protein that functions as a negative regulator of the receptor tyrosine-kinase ERBB2 [24]. Although TOB1 has been reported to play an important regulatory role in tumor cell proliferation and apoptosis, its role in host antiviral response remains unclear [25–27].

**Fig 2:**
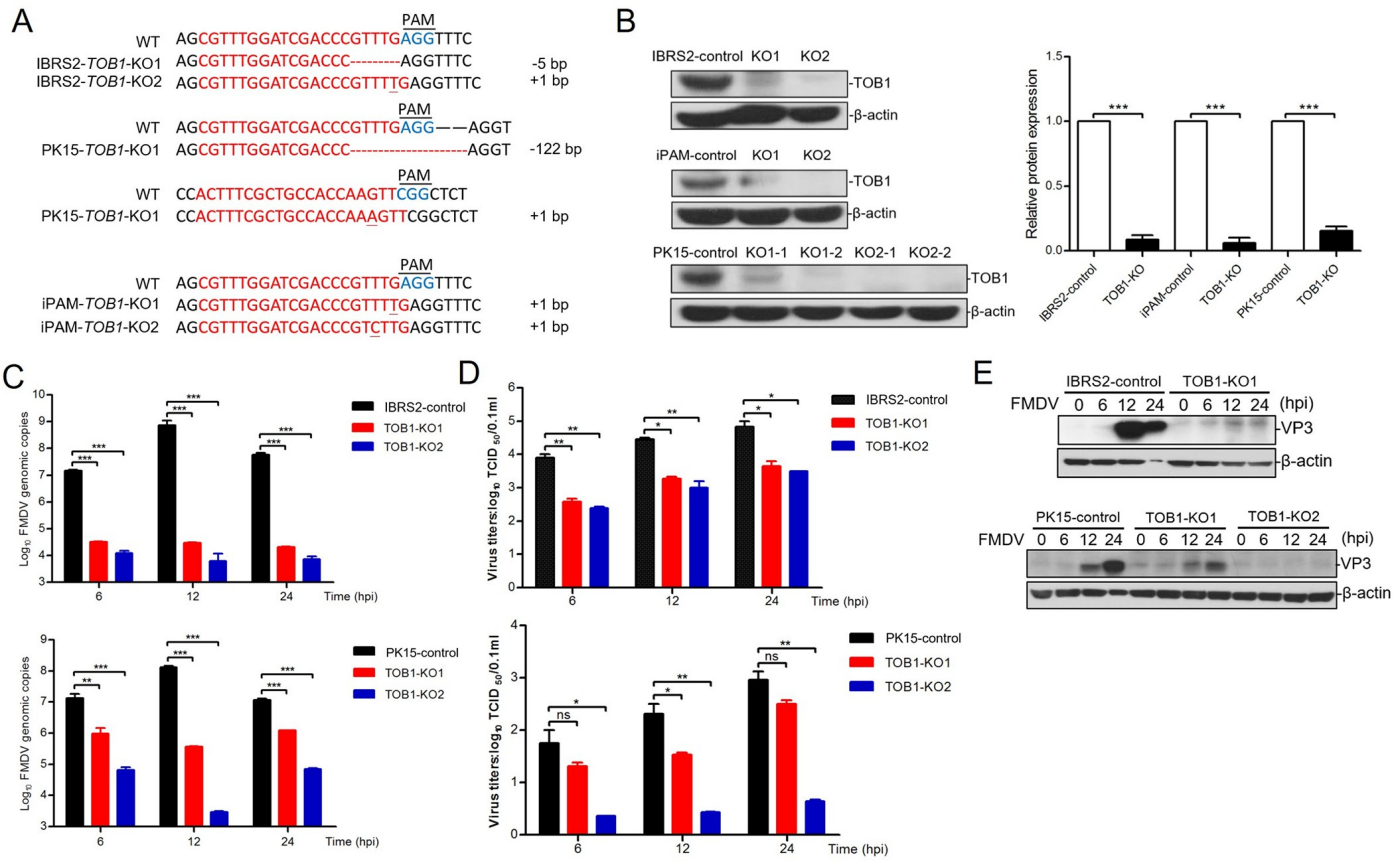
TOB1-knockout significantly inhibits FMDV replication in IBRS-2, PK-15, and iPAM cells. **A** Alignment of the nucleic acid sequences of clonal knockout cells of TOB1 with wild-type cells. sgRNA targeting sites are highlighted in red. The red characters “-” indicate the deleted bases in the knockout cells. Protospacer adjacent motif (PAM) sites are indicated in blue. **B** Protein levels of TOB1 in TOB1-knockout cells were detected by Western blot (left). Western blot results were quantitatively analyzed by image J software (right). **C** Absolute quantitative real-time PCR for determination of FMDV RNA copies number in TOB1-knockout IBRS-2 and PK-15 cells with FMDV infection at 0.1 MOI for 6, 12, and 24 h. **D, E** TOB1-knockout IBRS-2 and PK-15 cells were infected with equal amounts of FMDV (MOI of 0.1) for 6, 12, and 24 h, the viral titers were measured by TCID_50_ assay (D); the expression levels of viral proteins were detected by Western blot (E). KO1 and KO2, two monoclonal cell lines with different knockout types of IBRS-2, PK-15, and iPAM cells. Data shown includes technical replicates from a single experiment and is representative of three independent experiments (C). Data are represented as means ± S.D.; *P < 0.05; **P < 0.01; ***P < 0.001; ns, no significant. *P* values were determined by two-sided Student’s *t*-test.

To explore the role of TOB1 in FMDV infection, we constructed several *TOB1*-knockout FMDV-susceptible cell lines (IBRS-2, PK-15, and iPAM) using CRISPR/Cas9. Sanger sequencing confirmed that each of these knockout cell lines had several nucleotide insertions or deletions predicted to cause a frameshift mutation in the coding regions of the targeted gene (Fig 2A). Moreover, we verified the absence of TOB1 protein in the above three knockout cell lines using Western blot analysis (Fig 2B).

After 24 hours of FMDV infection, *TOB1*-knockout IBRS-2 cells showed lower cytopathic effect (CPE) and no significant changes in cell viability (S2A and S2D Fig). We quantified the FMDV genome copy numbers in *TOB1*-knockout and control cells at 6, 12, and 24 hours post-infection (hpi) using absolute quantitative PCR analysis targeting the 3D gene of FMDV. FMDV RNA was significantly reduced in *TOB1*-knockout IBRS-2 and PK-15 cells compared to control cells (Figs 2C and S2B). Similar results were observed in *TOB1*-knockout iPAM cells (S2E Fig). Immunofluorescence assays showed that the expression of FMDV-encoded VP3 protein in *TOB1*-knockout IBRS-2 cell lines was modestly reduced or undetectable during FMDV infection (S2C Fig). We conducted virus TCID_50_ assays for determination of viral load in *TOB1*-knockout IBRS-2 and PK-15 cell lines which were infected with FMDV at a multiplicity of infection (MOI) of 0.1 (Fig 2D). FMDV-infected *TOB1*-knockout cell lines of IBRS-2 and PK-15 possessed substantially reduced levels of viral VP3 protein as determined by Western blot (Fig 2E). These results suggest that TOB1 indeed plays an important role in FMDV infection.

### ISGs significantly upregulated in *TOB1*-knockout cells

The knockout of *TOB1* gene transformed FMDV-sensitive cells into FMDV-resistant cells, particularly in IBRS-2 cells. To investigate the role of TOB1 in viral infection, we used transcriptome sequencing to identify significantly differentially expressed genes between control and *TOB1*-knockout IBRS-2 cells. We also conducted gene ontology (GO) and kyoto encyclopedia of genes and genomes (KEGG) analyses to explore the molecular function, biological processes, and cell composition that TOB1 may participate in.

**Fig 3:**
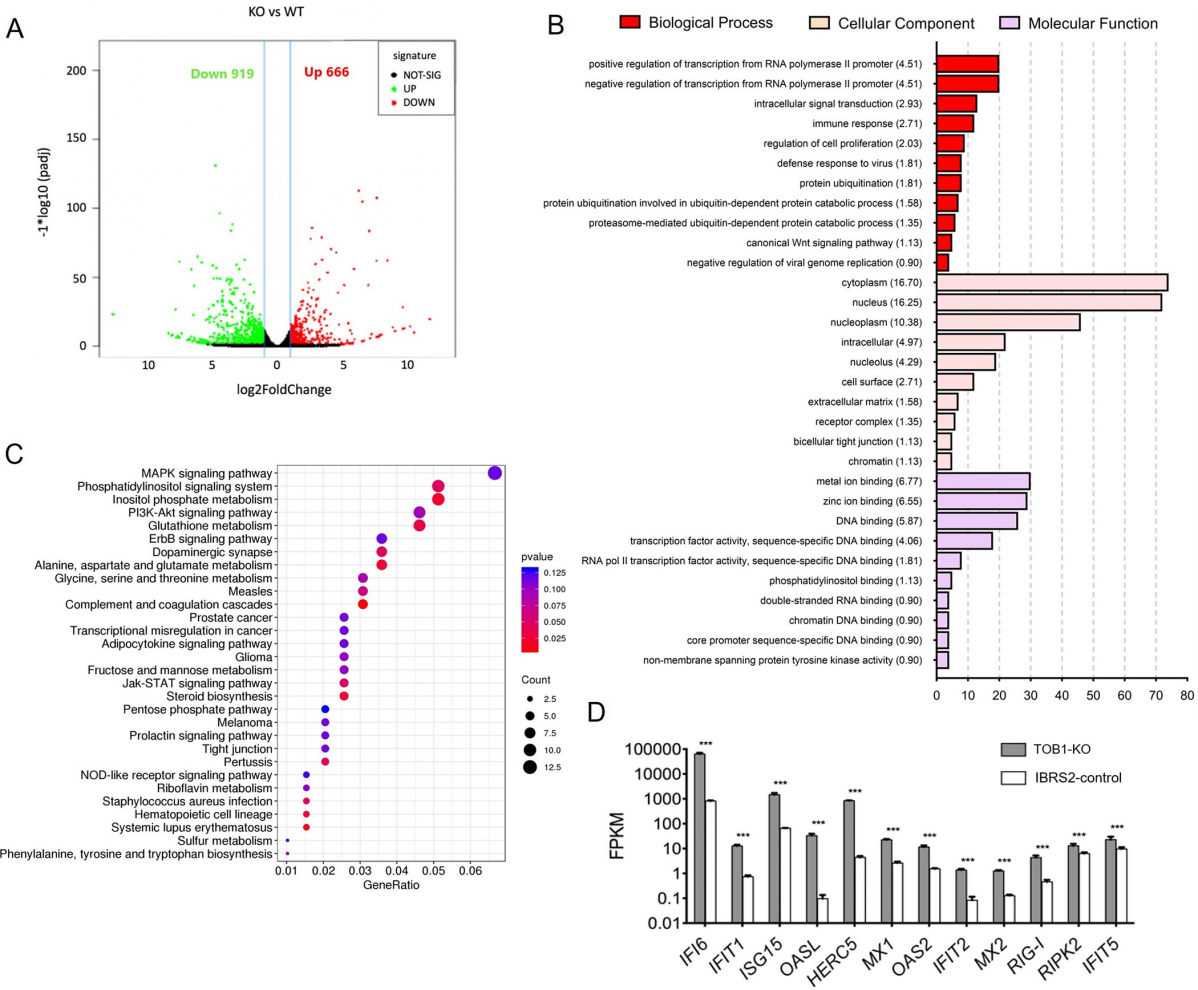
Comparison of RNA-seq results between IBRS-2 control and TOB1-knockout cells. **A** Volcano plots of DEGs (FDR<0.05 and log2^FC^>1) between control and TOB1-knockout IBRS-2 cells. The red dots represent the up-regulated DEGs and the green dots represent the down-regulated DEGs. The black dots indicate no significant difference. DEGs, differentially expressed genes. **B** Gene ontology (GO) function enrichment analysis of DEGs between control and TOB1-knockout IBRS-2 cells. **C** KEGG pathway analysis for DEGs between IBRS-2 control and TOB1-knockout cells. The color and the size of the dots represent the fold changes and the number of enriched DEGs, respectively. **D** A total of 12 DEGs involved in IFN pathway were selected and displayed in the column chart. FPKM, expected number of Fragments Per Kilobase of transcript sequence per Millions base pairs sequenced. Data shown includes technical replicates from a single experiment and is representative of three independent experiments (D). Data are represented as means ± S.D.; *P < 0.05; **P < 0.01; ***P < 0.001; ns, no significant. *P* values were determined by two-sided Student’s *t*-test.

We selected genes with a false discovery rate (FDR) less than 0.05 as significantly differentially expressed genes based on their expression levels. We found 1,585 significantly differentially expressed genes in *TOB1*-knockout cells, of which 919 genes were up-regulated and 666 genes were down-regulated, compared to control IBRS-2 cells (Fig 3A). As shown in S3A Fig, the three samples of control IBRS-2 cells (WT1, WT2, and WT3) clustered together, while the three samples of *TOB1*-knockout IBRS-2 cells (KO1, KO2, and KO3) formed another distinct group.

We performed GO and KEGG analyses to cluster the differentially expressed genes into categories such as molecular function, biological process, cell composition, and signal transduction pathway. *TOB1*-knockout IBRS-2 cells showed significantly up-regulated genes in biological processes such as cell proliferation, immune response, antiviral response, and inhibition of viral genome replication (Fig 3B). KEGG pathway analysis of the differentially expressed genes revealed upregulation of amino acid metabolism and JAK-STAT signaling pathways, while MAPK and ERBB signaling pathways were downregulated in *TOB1*-knockout cells (Fig 3C). Notably, several typical interferon stimulating genes (ISGs) and important antiviral effector genes, including *IFIT1*, *ISG15*, *MX1*, *OAS2*, and *IFIT5*, were significantly up-regulated in the differentially expressed genes (Fig 3D). These results suggested that *TOB1*-knockout significantly altered innate immune regulation of host antiviral responses, cell metabolism, and EGFR pathway regulation.

**Fig 4:**
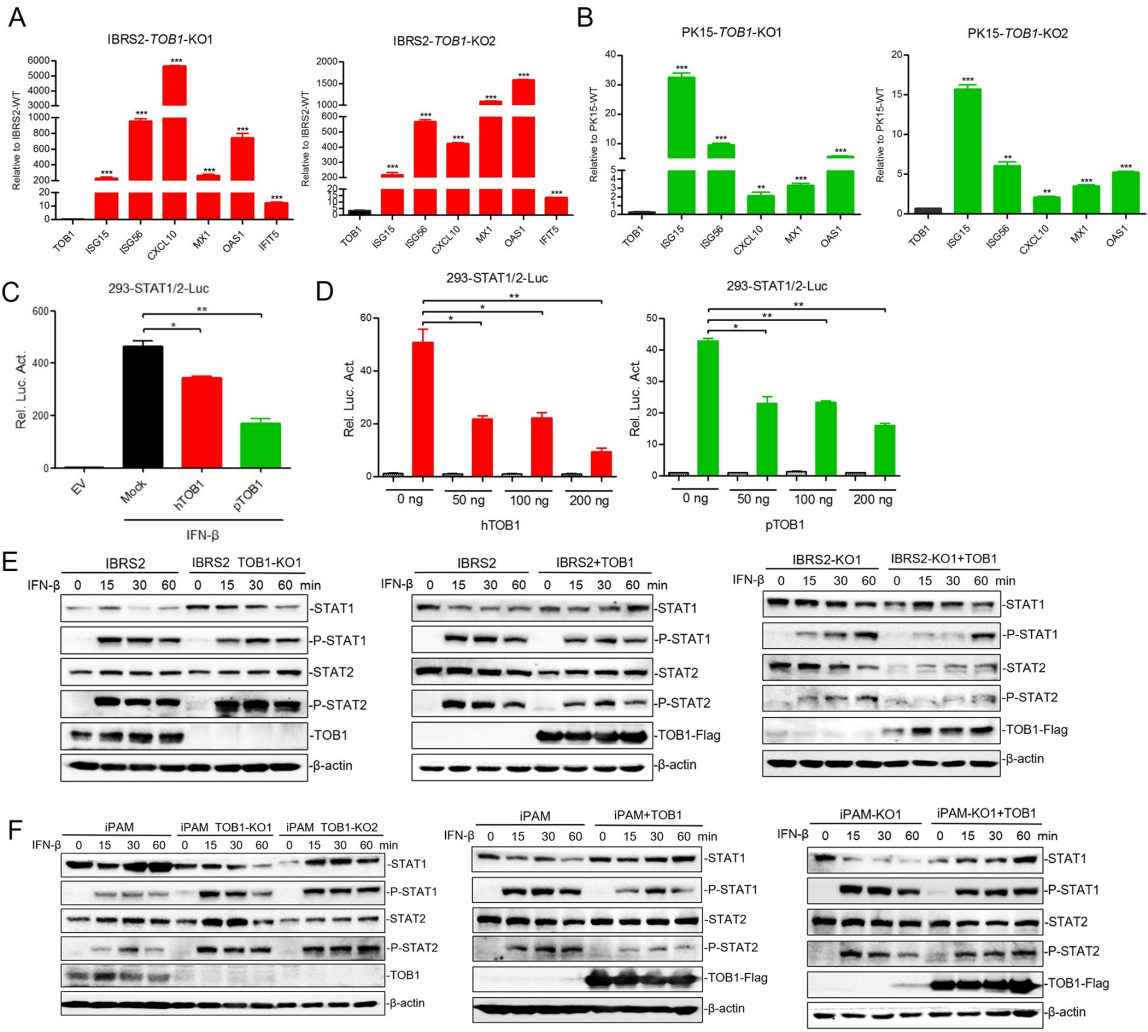
ISGs and the phosphorylation levels of STAT1/2 upregulated in TOB1-knockout cells. **A, B** The transcriptional levels of ISGs in TOB1-knockout IBRS-2 (A) and PK-15 (B) cells were measured by qPCR. **C, D** HEK293T cells were transfected with an empty vector, hTOB1-Flag or pTOB1-Flag plasmid (100 ng) (C) and hTOB1-Flag or pTOB1-Flag plasmid (0, 50, 100, or 200 ng) (D), and STAT1/2 promoter-driven luciferase reporter plasmid together with an internal control pRL-TK reporter plasmid for 24 h. Cells were stimulated with IFN-β for another 12 h, and whole-cell lysates were collected for measurements of luciferase activity. **E, F** The protein and phosphorylation of STAT1/2 in TOB1-knockout IBRS-2 and iPAM cells (left), TOB1-overexpressed cells (middle), and TOB1-knockout cells with TOB1 plasmid rescued (right) were detected by Western blot at 0 min, 15 min, 30 min and 60 min treated with IFN-β. EV, transfected with empty vector; Mock, transfected with empty vector and treated with IFN-β; hTOB1, human TOB1 expressing plasmid; pTOB1, pig TOB1 expressing plasmid. Data shown includes technical replicates from a single experiment and is representative of two independent experiments (A, B, C, D). Data are represented as means ± S.D.; *P < 0.05; **P < 0.01; ***P < 0.001; ns, no significant. *P* values were determined by two-sided Student’s *t*-test.

To validate that *TOB1*-knockout leads to the upregulation of numerous ISGs, we examined the transcriptional levels of ISGs in *TOB1*-knockout IBRS-2 and PK-15 cells. We found a significant increase in the expression of several antiviral-related ISGs, including *ISG15*, *ISG56*, *CXCL10*, *MX1*, and *OAS1*, especially in *TOB1*-knockout IBRS-2 cells (Fig 4A and 4B). We also observed a significant expressional increase in ISGs in *TOB1*-knockout iPAM cells (S3B Fig). Transcription of ISGs in cells is primarily regulated by the JAK-STAT signaling pathway, the production of upstream IFN-I and its binding to IFN receptor is a key factor in activating this pathway [28]. Thus, our study first explored the effect of TOB1 on the JAK-STAT signaling pathway, and we found that the overexpression of TOB1 could inhibit IFN-β-triggered STAT1/2 promoter activity in a dose-dependent manner (Fig 4C and 4D).

With binding to its receptor, type I IFN phosphorylates the downstream TYK2 and JAK1. Both activated JAK1 and TYK2 further phosphorylate the downstream tyrosine residues of STAT1/2 [29]. STAT1 and STAT2 combine to form a dimer, and then recruit IRF9 and transport it to the nucleus to regulate the expression of abundant antiviral genes [30]. Thus, the expression and phosphorylation activation of key molecules in the JAK-STAT pathway is critical for the signaling of this pathway. Under the stimulation of IFN-β, *TOB1*-knockout resulted in a significant increase in the mRNA levels of *STAT1* and *IRF9* (S3E Fig). The protein and phosphorylation of STAT1/2 in *TOB1*-knockout IBRS-2/iPAM cells and *TOB1* overexpression cells were detected by Western blot at 0 min, 15 min, 30 min and 60 min treated with IFN-β. *TOB1*-knockout resulted in slightly increased STAT1 protein levels and STAT2 phosphorylation. Overexpression of *TOB1* inhibited the phosphorylation of STAT1/2. Similarly, the phosphorylation of STAT1/2 was also inhibited in *TOB1*-knockout cells transformed with TOB1 expressing plasmid (Fig 4E). In iPAM cells, TOB1 also inhibited the STAT1/2 pathway, but the effect on STAT1 protein was inconsistent with that in IBRS-2 cells, which may be caused by cell type differences (Fig 4F). Taken together, the above results showed that TOB1 inhibited JAK-STAT signaling by inhibiting the phosphorylation of STAT1/2.

### *TOB1*-knockout potentiated the expression of RIG-I and MDA5

We observed that the knockout of *TOB1* gene resulted in increased protein level of IFN-β under poly(I:C) stimulation (S3D Fig). Moreover, the luciferase reporter assay showed that the overexpression of TOB1 inhibited SeV-triggered IFN-β promoter activity (S3C Fig). These results suggest that TOB1 may also play a role in the upstream of the IFN pathway.

**Fig 5:**
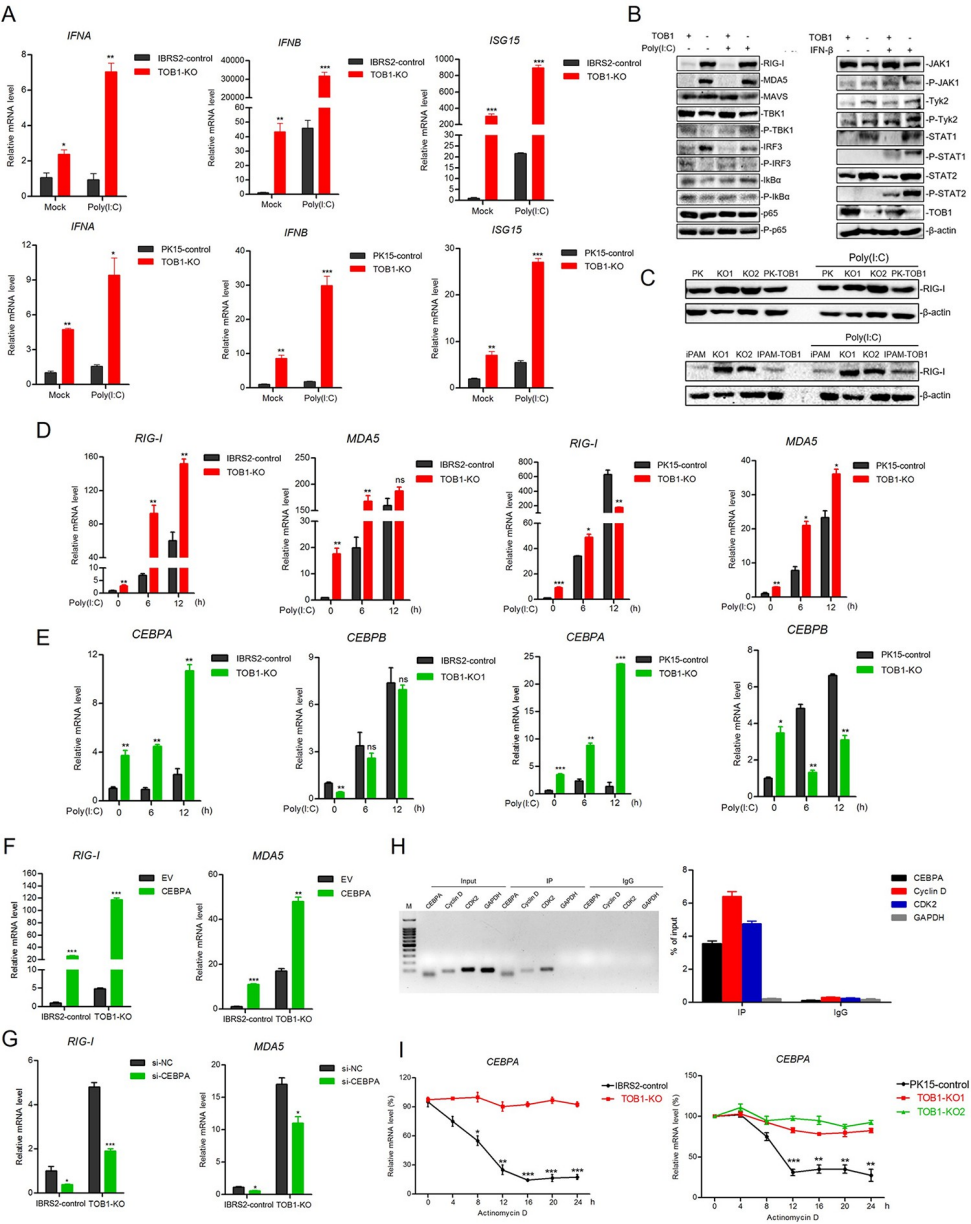
TOB1-knockout potentiated the expression of RIG-I and MDA5. **A** IBRS-2 and PK-15 control cells and TOB1-knockout cells were transfected with poly(I:C) for 12 h. The transcriptional levels of *IFNA*, *IFNB*, and *ISG15* were measured by qPCR, respectively. **B** The key nodal molecules including RIG-I, MDA5, and JAK-STAT pathway were detected by Western blot, under either poly(I:C) or IFN-β-stimulated in TOB1-knockout IBRS-2 cells. **C** TOB1-knockout PK-15 and iPAM cells were transfected with poly(I:C) for 12 h. The protein level of RIG-I was measured by Western blot. **D, E** IBRS-2 and PK-15 control cells and TOB1-knockout cells were transfected with poly(I:C) for 0 h, 6 h, and 12 h. The mRNA level of *RIG-I* and *MDA5* (D), *CEBPA* and *CEBPB* (E) were measured by qPCR. **F, G** IBRS-2 control cells and TOB1-knockout cells were transfected with pRK-CEBPA-HA or pRK-HA plasmid (F), si-CEBPA or si-NC (G) for 24 h. The mRNA level of *RIG-I* and *MDA5* were measured by qPCR. **H** The binding of TOB1 to *CEBPA* mRNA was detected by RNA immunoprecipitation assay. The RNA obtained from RNA immunoprecipitation was reverse transcribed and subjected to PCR amplification and detected by agarose gel electrophoresis (left), and the enrichment efficiency of different genes is detected by qPCR (right). **I** The mRNA levels of *CEBPA* were examined with adding Actinomycin D for 0, 4, 8, 12, 16, 20, 24 h by qPCR in IBRS-2 and PK-15 control cells and TOB1-knockout cells. CEBPA/B, CCAAT enhancer binding protein alpha/beta; si-NC, si-negative control; CDK2, cyclin dependent kinase 2. Data shown includes technical replicates from a single experiment and is representative of three independent experiments (A, D, E). Data are represented as means ± S.D.; *P < 0.05; **P < 0.01; ***P < 0.001; ns, no significant. *P* values were determined by two-sided Student’s *t*-test.

*TOB1*-knockout cells potentiated poly(I:C)-induced IFN expression compared to control cells (Fig 5A). The innate immune pathway against FMDV infection in cells begins with the recognition of viral RNA by pattern recognition receptors (PRRs), and then triggers the expression of downstream antiviral genes through a series of signal transduction [31]. To explore the role of TOB1 in innate immune pathways, we detected expression of the key nodal molecules including RIG-I and MDA5 by Western blot, under either poly(I:C) or IFN-β-stimulated. *TOB1*-knockout resulted in increases in RIG-I and MDA5 protein levels, as well as STAT1/2 phosphorylation levels downstream of the IFN pathway in IBRS-2 cells (Fig 5B). Similar results were observed in *TOB1*-knockout PK-15 and iPAM cells (Fig 5C). Using qPCR, we also found that *TOB1*-knockout resulted in elevated *RIG-I* and *MDA5* mRNA levels (Fig 5D).

Several transcription factors have been reported to be involved in the regulation of RIG-I and MDA5 expression. CEBPA was one of the few transcription factors identified in our transcriptome analysis capable of regulating RIG-I and MDA5 expression, and we found that CEBPA expression was significantly upregulated in *TOB1*-knockout cells. Specifically, CEBPA, but not CEBPB, expression was significantly increased in *TOB1*-knockout cells, especially under poly(I:C) stimulation (Fig 5E). We confirmed the effect of CEBPA on RIG-I and MDA5 expression by overexpressing or knocking down CEBPA in IBRS-2 and PK-15 cells (S4A Fig). Ectopic expression of CEBPA promoted RIG-I and MDA5 expression, whereas knockdown of CEBPA suppressed their expression (Fig 5F and 5G). Moreover, ectopic expression of CEBPA promoted the mRNA expression of *IFNB*, *ISG15*, and *ISG56* (S4C Fig).

TOB1 has been reported to regulate the transcription of specific genes by binding to their mRNA transcripts, and affecting their mRNA stability [32,33]. Therefore, we next explored the binding of TOB1 to CEBPA mRNA and its effect on CEBPA stability. The RNA immunoprecipitation assay showed that TOB1 could bind to CEBPA mRNA (Fig 5H). The mRNA levels of CEBPA were examined at different time points by adding Actinomycin D to inhibit gene transcription. Compared with *TOB1*-knockout cells, the *CEBPA* mRNA level in control cells was significantly reduced, indicating that TOB1 could regulate *CEBPA* expression at the mRNA level (Fig 5I). Of note, TOB1 has a more significant inhibitory effect on CEBPA in IBRS-2 cells, rather in PK-15 cells. Moreover, we observed that the knockdown of CEBPA significantly promoted FMDV infection in *TOB1*-knockout IBRS-2 cells (S4B Fig), suggesting that the antiviral effect of *TOB1*-knockout is mediated by CEBPA. The knockdown of RIG-I inhibits the IFN pathway and the ectopic expression of ISG15 and MX1 inhibits the infection of FMDV (S4D–S4F Fig). Taken together, our results suggest that *TOB1*-knockout leads to an elevated expression of CEBPA, especially in IBRS-2 cells, which positively regulates RIG-I and MDA5 expression and activates the IFN pathway.

### *TOB1*-knockout inhibits FMDV entry by inhibiting EGFR pathway

In immunofluorescence assays of *TOB1*-knockout IBRS-2 cells infected with FMDV, viral particles were hardly detected during the early stages of infection (S2C Fig). Moreover, the viral RNA levels in *TOB1*-knockout IBRS-2, PK-15, and iPAM cells were lower than those in control cells at 6 hpi (Figs 2C and S2E), suggesting that *TOB1* deletion may play important roles not only in the innate immune pathway but also in virus entry. Furthermore, KEGG pathway analysis of transcriptome data (Fig 3D) revealed that *TOB1*-knockout led to a significant down-regulation of the ERBB2 (also known as EGFR2) signaling pathway. In recent years, increasing evidence has shown that viruses can interact with EGFR and modulate its activity to promote virus entry, replication, or evasion of host immune response [34,35]. For instance, ERBB2 can be a potential target for Ebola virus (EBOV) inhibitors and play a crucial role in EBOV infection [36]. Therefore, as TOB1 is a regulatory protein of ERBB2, it is intriguing to investigate whether the antiviral phenotype induced by *TOB1*-knockout is also related to the process of virus entry.

**Fig 6:**
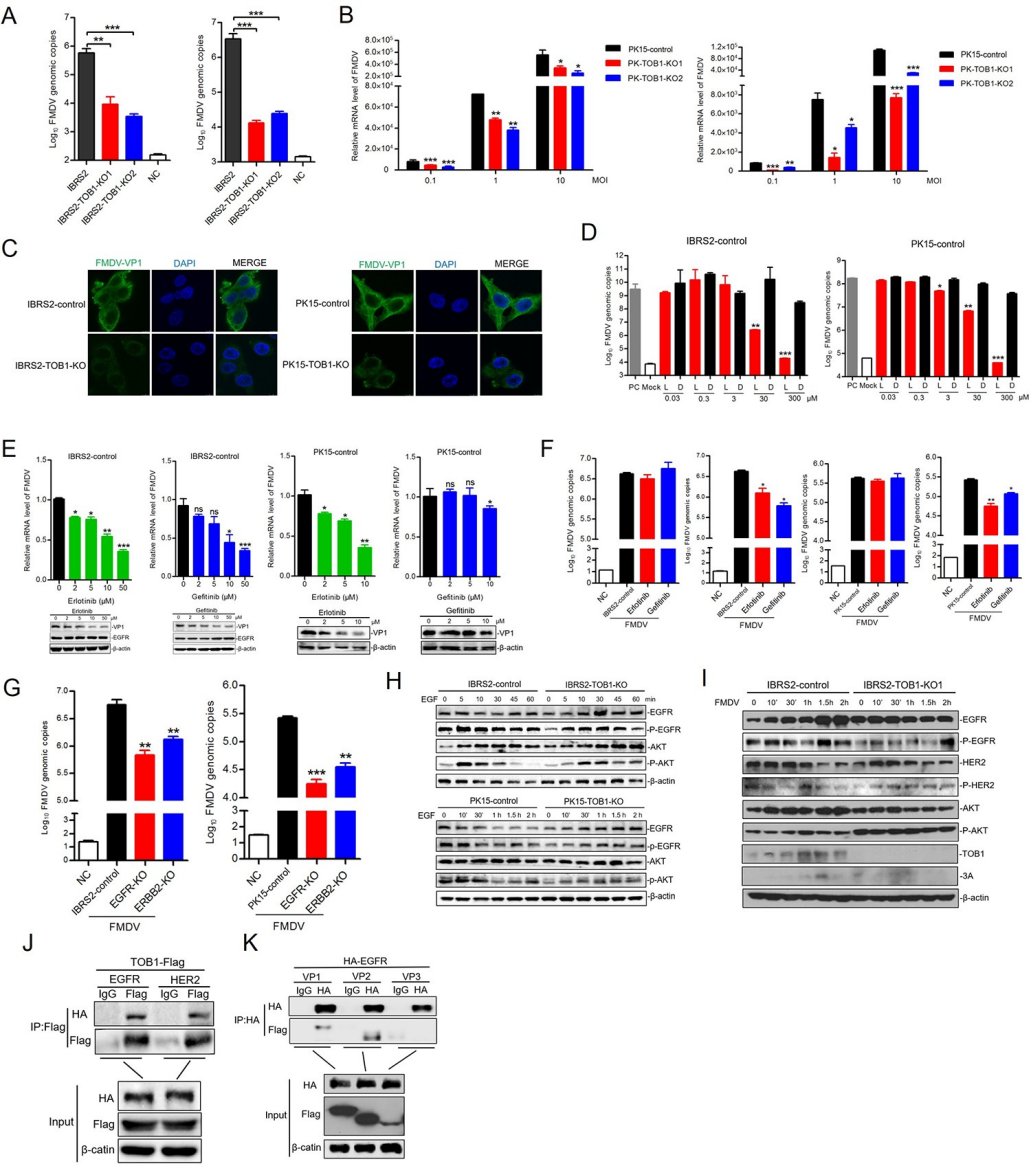
TOB1-knockout inhibits FMDV entry by inhibiting EGFR pathway. **A** IBRS-2 Control and TOB1-knockout IBRS-2 cells were infected with FMDV for 1 h at 4°C (left) or 30 min at 37°C (right). The FMDV RNA copies number were detected by absolute quantitative real-time PCR. NC (left panel), FMDV was incubated with neutralizing antibodies and was then used to infect cells. FMDV positive serum was provided by the Foot-and-Mouth Disease Reference Laboratory. NC (right panel), cells were infected by FMDV after Chlorpromazine (CPZ)-treatment. **B** PK-15 control and TOB1-knockout cells were infected with FMDV (MOI of 0.1, 1 and 10) for 1 h at 4°C (left) or 30 min at 37°C (right). The replication levels of FMDV were quantified by qPCR. **C** TOB1-knockout cells and control cells were infected with FMDV (MOI of 10) for 30 min at 37°C. The samples were subjected to immunofluorescence using anti-VP1 antibody. **D** IBRS-2 and PK-15 control cells were treated with EGFR/ERBB2 tyrosine kinase domain inhibitor (Lapatinib) or DMSO at 0.03, 0.3, 3, 30, 300 μM for 24 h. Then, cells were infected with FMDV for 12 h at 0.01 MOI. The FMDV RNA copies number were detected by absolute quantitative real-time PCR. PC, positive control. Mock, uninfected group. L, Lapatinib. D, DMSO. **E** IBRS-2 and PK-15 control cells were treated with EGFR specific inhibitors (Erlotinib and Gefitinib) or DMSO at 0, 2, 5, 10 μM for 24 h. Then, cells were infected with FMDV for 12 h at 0.01 MOI. The replication levels of FMDV were measured by qPCR. **F** IBRS-2 and PK-15 control cells were treated with EGFR specific inhibitors (Erlotinib and Gefitinib) at 10 μM for 24 h. Then, cells were infected with FMDV for 1 h at 4°C (the first and third sub-panels of Fig 6F) or 30 min at 37°C (the second and fourth sub-panels of Fig 6F). Absolute quantitative real-time PCR was performed to detect the FMDV RNA copy numbers. **G** IBRS-2 and PK-15 control cells, EGFR and ERBB2-knockout cells were infected with FMDV for 30 min at 37°C. Absolute quantitative real-time PCR was performed to detect the FMDV RNA copies number. **H** IBRS-2 control and TOB1-knockout cells were treated with EGF for 0, 5, 10, 30, 45, and 60 min at 10 μM (up) and PK-15 control and TOB1-knockout cells were treated with EGF for 0, 10 min, 30 min, 1 h, 1.5 h, and 2 h at 10 μM (down). Western blot was performed using anti-EGFR, anti-pEGFR, anti-AKT, and anti-pAKT antibodies. **I** Control and TOB1-knockout IBRS-2 cells were infected with FMDV at 0.1 MOI for 0–2 h. The samples were subjected to Western blot for EGFR pathway expression and activation. **J** HEK293T cells were co-transfected with pPB-EF1α-TOB1-Flag and pRK-EGFR-HA or pRK-ERBB2-HA. The interactions of TOB1 and EGFR or ERBB2 were examined using co-immunoprecipitation. **K** HEK293T cells were co-transfected with pRK-EGFR-HA and VP1, VP2, or VP3. The interactions of EGFR and FMDV viral proteins were examined using Co-immunoprecipitation. Data shown includes technical replicates from a single experiment and is representative of three independent experiments (A, E, F, G). Data are represented as means ± S.D.; *P < 0.05; **P < 0.01; ***P < 0.001; ns, no significant. *P* values were determined by two-sided Student’s *t*-test.

To verify the role of TOB1 in the FMDV entry process, we quantified FMDV RNA copies in FMDV-infected *TOB1*-knockout IBRS-2 cells during 1 h at 4°C or 30 min at 37°C to examine virus adsorption and internalization. The results showed that *TOB1*-knockout inhibited FMDV adsorption and internalization (Fig 6A). We also examined the adsorption and internalization of FMDV in *TOB1*-knockout PK-15 and iPAM cells at MOI of 0.1, 1, and 10 by immunofluorescence and quantification. Consistent with the results in IBRS-2 cells, *TOB1*-knockout inhibited the adsorption and internalization of FMDV (Figs 6B and S5A and S5B). In addition, we also found that *TOB1*-knockout inhibited the internalization of FMDV compared with FMDV-infected control cells by immunofluorescence (Fig 6C). These findings suggest that *TOB1*-knockout could inhibit FMDV entry in multiple cell types, indicating that it also plays a crucial role in the virus entry.

*TOB1*-knockout inhibited the infection of FMDV, presumably through the EGFR/ERBB2 signaling pathway. The activation of this pathway requires the tyrosine kinase domain. Thus, we treated IBRS-2, PK-15, and iPAM cells with the EGFR/ERBB2 tyrosine kinase domain inhibitor Lapatinib and measured their effects on FMDV infection. FMDV RNA copy numbers were significantly reduced in cells treated with 30 μM Lapatinib, indicating that inhibiting EGFR/ERBB2 tyrosine kinase activity and activation of this pathway can inhibit virus infection (Figs 6D and S5V). To confirm that the inhibition of EGFR kinase activity specifically affected FMDV infection, we treated IBRS-2 and PK-15 cells with EGFR-specific inhibitors Erlotinib and Gefitinib (S5E Fig), and then infected them with FMDV, then quantified viral RNA and protein. Both inhibitors inhibited FMDV infection in IBRS-2 and PK-15 cells, indicating that specific inhibition of EGFR kinase activity can inhibit FMDV infection (Fig 6E).

The inhibition of the EGFR pathway can prevent the final infection of FMDV. However, it is unclear whether it plays a role in the early stages of virus infection, such as adsorption and internalization. To investigate this, we examined the effects of Erlotinib and Gefitinib on FMDV adsorption and internalization in IBRS-2 and PK-15 cells. Our results showed that Erlotinib and Gefitinib inhibited the kinase activity of EGFR and the internalization of FMDV, but not its adsorption (Fig 6F). The knockdown of EGFR or ERBB2 inhibited the internalization of FMDV in IBRS-2 cells (S5F and S5G Fig). Moreover, we also found that the knockout of EGFR or ERBB2 inhibited the internalization and infection of FMDV (Figs 6G and S5H). These findings suggest that the reduce of EGFR/ERBB2 protein level and the inhibition of their phosphorylation level can also inhibit FMDV entry.

To further explore the effect of TOB1 on EGFR/ERBB2-mediated FMDV entry, we investigated the EGFR pathway in IBRS-2 and PK-15 cells. We stimulated the cells with epidermal growth factor (EGF), which can activate the EGFR pathway and transmit phosphorylation signals downstream to phosphorylate AKT. In control cells, EGF stimulation caused phosphorylation of EGFR and AKT in the early stage, but the activation of this pathway was inhibited in *TOB1*-knockout cells (Fig 6H). Moreover, EGF treatment enhanced FMDV infection in *TOB1*-knockout IBRS-2 cells (S5D Fig). Ectopic expression of EGFR also promoted FMDV infection, indicating that TOB1 affects FMDV infection by regulating the EGFR pathway (S5I Fig). We further found that *TOB1*-knockout inhibited the activation of the EGFR pathway at 0 min, 10 min, 30 min, 1 h, 1.5 h, and 2 h of the early stage of FMDV infection (Fig 6I). Finally, we confirmed that TOB1 can interact with EGFR and ERBB2, and EGFR can interact with VP1 and VP2 of FMDV (Fig 6J and 6K). In summary, our findings suggest that in IBRS-2, PK-15, and iPAM cells, the deletion of TOB1 inhibits the phosphorylation of EGFR and the downstream signal transduction pathway, thereby inhibiting the early entry of FMDV. Notably, the significant increase in ISGs caused by TOB1 deficiency is the main factor in inhibiting viral infection in IBRS-2 cells, while TOB1 deletion inhibiting FMDV infection via the EGFR pathway, is common in IBRS-2, PK-15, and iPAM cells.

### *TOB1* deletion prevents FMDV infection *in vivo*

**Fig 7:**
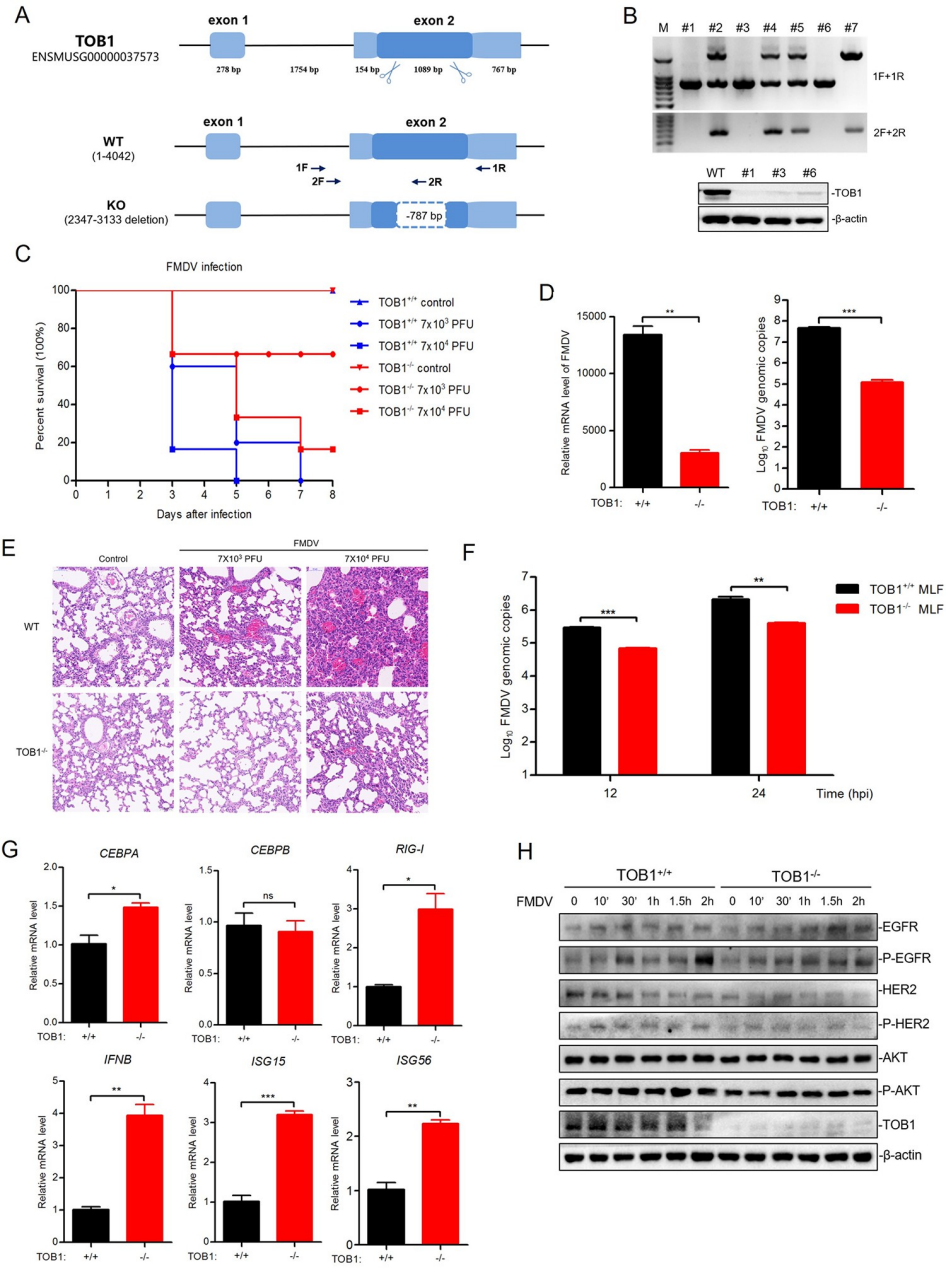
TOB1 deletion prevents FMDV infection in vivo. **A** Knockout schematic representation position of the sequence for TOB1 in *TOB1*^-/-^ mice. The mouse genome of TOB1 contained two exons. CRISPR/Cas9 technology was used to design a targeted vector to exon 2. **B** Expression and protein levels of TOB1 in *TOB1*^-/-^ and *TOB1*^+/+^ mice were detected by PCR amplification and Western blot to determine TOB1 knockout efficiency. **C** Three-day-old *TOB1*^-/-^ and *TOB1*^+/+^ (n = 6 in each group) suckling mice were used and divided into three groups. One group was subcutaneously injected with equal amounts of PBS, and the other two groups were injected subcutaneously with two concentrations of FMDV (7×10^3^ and 7×10^4^ PFU). The survival rate was monitored every 24 h. One of the *TOB1*^+/+^ mice group was died before injected with 7×10^3^ PFU FMDV. **D** Three-day-old *TOB1*^-/-^ and *TOB1*^+/+^ (n = 4 in each group) mice were used and subcutaneously injected with equal amounts of PBS and FMDV (7×10^4^ PFU) and euthanized at 72 h after FMDV infection. Samples from FMDV-infected mouse were detected by absolute quantitative real-time PCR individually. **E** The lung tissue in *TOB1*^-/-^ and *TOB1*^+/+^ mice injected with equal amounts of PBS and FMDV (7×10^4^ PFU) and euthanized at 72 h after FMDV infection were subjected to HE staining. **F** The MLF cells from *TOB1*^-/-^ and *TOB1*^+/+^ mice were infected with FMDV of 0.1 MOI for 12 and 24 h. The FMDV RNA copies were measured by absolute quantitative real-time PCR. **G** The expression of *CEBPA*, *CEBPB*, *RIG-I*, *IFNB*, *ISG15*, and *ISG56* were detected by qPCR in the MLF cells from *TOB1*^-/-^ and *TOB1*^+/+^ mice. **H** The MLF cells from *TOB1*^-/-^ and *TOB1*^+/+^ mice were infected with FMDV of 0.1 MOI for 0, 10 min, 30 min, 1 h, 1.5 h, and 2 h. The samples were subjected to Western blot for EGFR pathway expression and activation. Data shown includes technical replicates from a single experiment and is representative of three independent experiments (D, F, G). Data are represented as means ± S.D.; *P < 0.05; **P < 0.01; ***P < 0.001; ns, no significant. *P* values were determined by two-sided Student’s *t*-test.

TOB1 plays a critical role in FMDV infection *in vitro*, and its deletion can confer cells with a potent antiviral phenotype. To investigate whether the deletion of TOB1 also protects against FMDV challenge in mice, we used CRISPR/Cas9 to introduce mutations by non-homologous recombination repair in the second exon of the *TOB1* gene, resulting in a frameshift and functional loss of TOB1 protein. Compared with wild-type, the results showed that the *TOB1*-mutated gene copy has a deletion of 787 bp (Fig 7A). Furthermore, F1 mice were used for genotyping and TOB1 protein detection (Fig 7B).

We subcutaneously injected wild-type and *TOB1*-knockout mice with a high dose (7 × 10^4^ PFU) and low dose (7 × 10^3^ PFU) of FMDV, respectively, and a control group was injected with PBS. Under low dose challenge, all wild-type mice died within 7 days, whereas 67% of *TOB1*-knockout mice survived, indicating a significant protective effect (Fig 7C). At 72 h post-FMDV infection, the replication level and copy numbers of FMDV RNA were lower in *TOB1*-knockout mice compared to wild-type mice (Fig 7D). Furthermore, we observed significant pathological changes and inflammatory reactions in the lung tissues of wild-type mice during FMDV infection, while no significant changes were observed in *TOB1*-knockout mice (Fig 7E). We also found that the deletion of TOB1 resulted in a decrease in FMDV infection in mouse lung fibroblast (MLF) cells (Fig 7F). In control MLF cells, the deletion of TOB1 increased CEBPA and RIG-I mRNA levels, as well as IFN-β and downstream ISGs (Fig 7G), indicating that the deletion of TOB1 enhances innate immune signaling. Furthermore, we confirmed that the deletion of TOB1 inhibits EGFR phosphorylation activation at the 2 h time point of FMDV infection in MLF cells (Fig 7H). In summary, these results indicate that TOB1 also inhibits FMDV infection *in vivo*. The deletion of TOB1 confers cells and mice with a potent antiviral phenotype, which is attributed to the enhancement of innate immune signaling and the inhibition of the EGFR pathway in the early stages of FMDV infection.

## Discussion

The main function of TOB1 is to inhibit tumor cell proliferation and promote tumor cell apoptosis through multiple signaling pathways [26,27]. More recently, it has been discovered that TOB1 is involved in the immunomodulatory process. For instance, TOB1 can influence the expression of IL-2, a key cytokine in T cell activation, and inhibit T cell activation by interacting with the SMAD family of transcription factors [37]. TOB1 can also interact with CAF1, which recruits the CCR4-CAF1 complex to enhance deacetylation, thereby degrading target mRNA and regulating IL-2 production in T cells [38]. This study has uncovered a new function of TOB1 in the host antiviral response. In summary, deletion of TOB1 enhances the RIG-I/MDA5-mediated host antiviral immune response and inhibits viral entry mediated by the EGFR/ERBB2 pathway, ultimately suppressing FMDV infection.

During virus infection, the host innate immune pathway produces IFN and downstream antiviral cytokines, which are essential for inhibiting viral replication. In control IBRS-2 cells, low levels of IFN and ISGs are expressed without stimulation, whereas knockout of *TOB1* significantly increases the expression of IFN-β and ISGs. *TOB1*-knockout in PK-15 and iPAM cells also increases ISGs, but the effect is less pronounced than in IBRS-2 cells, possibly due to differences in cell type. In addition, we have intentionally tested the IBRS-2 and PK-15 cell lines used in our study and found that these cells were not contaminated by classical swine fever virus (data not shown). Among the three cell lines, the anti-FMDV infection phenotype of *TOB1*-knockout in IBRS-2 cells is the most significant, likely due to the high expression of ISGs. Previous studies have shown that during Seneca virus A (SVA) infection, the innate immune response-related pathway is more effectively activated in PK-15 cells than in IBRS-2 cells. Both IBRS-2 and PK-15 cells have a well-balanced JAK-STAT signaling pathway, but signal transduction from TBK1 to IRF3 in the RLR signaling pathway of IBRS-2 cells is blocked, leading to a different innate immune response and virus replication in the two cell lines [39]. We investigated the effect of TOB1 on the JAK-STAT signaling pathway and found that ectopic expression of TOB1 inhibits the phosphorylation of STAT1/2, thereby reducing the expression of downstream ISGs. *TOB1*-knockout in IBRS-2 and PK-15 cells led to an increase in IFN-β, especially in response to Sendai virus (SeV) infection or poly(I:C) stimulation, indicating that TOB1 also plays a negative regulatory role in the upstream of the IFN pathway. We further found that TOB1 acts on RIG-I and MDA5 through CEBPA to regulate the innate immune pathway. *TOB1*-knockout leads to increased protein levels of RIG-I and MDA5, directly enhancing the innate immune signal pathway and its downstream transmission, including the production of IFN and high expression of ISGs. A recent study has also reported that TOB1 negatively regulates IFN-β by inhibiting its transcription in macrophages by disrupting the binding of IRF3 and recruiting HDAC8 to the IFN-β1 promoter region [40].

The growth, proliferation, apoptosis, and differentiation of cells are typically influenced by the cellular and systemic environment. Cell membrane protein tyrosine kinase receptors play a crucial role in receiving extracellular signals and transmitting them into the cell. The EGFR/ERBB2 family signaling pathway consists of tyrosine kinase receptors such as EGFR1 (ERBB1, EGFR), EGFR2 (ERBB2), EGFR3 (ERBB3), and EGFR4 (ERBB4), which are present in the cell membrane. Ligands, such as EGF and TGF-α, can bind to the extracellular domain of EGFR receptor family proteins, leading to the formation of homologous or heterologous dimers that activate the whole pathway [41]. ERBB2 does not have a ligand that binds to it, but it can form a dimer with any other member of the EGFR family, making it the core of the EGFR family signaling. Upon dimer formation, the EGFR receptor family initiates multiple downstream signaling pathways, primarily the PI3K/AKT and Ras/Raf/MEK/MAPK signaling [42].

Due to the role of EGFR in endocytosis and cytoskeletal remodeling, several viruses can use the EGFR signaling pathway to enter host cells. For instance, the envelope protein of HCMV can interact with EGFR to induce signal transduction and entry into cells [43], while HSV-1 activates a similar EGFR-dependent signaling cascade to regulate F-actin remodeling and facilitate its entry into cells [44]. HCV and IAV also perform receptor-mediated endocytosis in coordination with EGFR activation. During HCV infection, EGFR colocalizes with CD81 to induce endocytosis, while IAV activates EGFR in a sialic acid-dependent manner [45]. In FMDV infection, macropinocytosis mediated by receptor tyrosine kinase promotes FMDV endocytosis into cells [46].

We found that *TOB1*-knockout inhibits viral entry, and the reduction of EGFR and ERBB2 expression and the inhibition of their kinase activity also significantly inhibit viral entry in the early stage of FMDV infection. Further experiments showed that TOB1 interacts with EGFR/ERBB2, and its deletion inhibits the phosphorylation activation of EGFR and downstream AKT signal transduction, thereby inhibiting early FMDV infection. However, the specific mechanism of the EGFR pathway in FMDV entry into cells requires further exploration. Additionally, although we found that TOB1 interacts with EGFR/ERBB2 and regulates EGFR phosphorylation activation, it is unclear how TOB1 regulates EGFR phosphorylation since it does not have kinase activity. Moreover, EGFR and ERBB2 often function as heterodimers, so further investigation is needed to determine how TOB1, EGFR, and ERBB2 interact with each other.

*TOB1*-knockout not only exhibits a significant antiviral phenotype at the cellular level, but also protects mice from lethal doses of FMDV challenge, suggesting that TOB1 has potential applications in molecular breeding for disease resistance. A representative study in animal disease resistance breeding is the gene editing of pigs to disrupt the PRRSV receptor CD163, which inhibits PRRSV infection without affecting the growth and development of pigs [47]. Since TOB1 is a tumor suppressor gene with an important anti-proliferative physiological function, complete deletion of TOB1 in pigs may lead to the spontaneous formation of multiple tumors, as reported in a mouse study [48]. Therefore, further exploration is needed to understand the interaction mechanism between TOB1 and viruses to enable an accurate gene editing, which does not affect the normal physiological function of TOB1, but hopefully generates an anti-FMDV phenotype in gene edited pigs.

## Materials and methods

### Ethics statement

All experiments involving animals and FMDV were conducted in strict accordance with good animal practice according to the Animal Ethics Procedures and Guidelines of the People’s Republic of China, and the study was approved by the Animal Ethics Committee of Lanzhou Veterinary Research Institute, Chinese Academy of Agricultural Sciences (approved number: No. LVRIAEC-2021-019, approved on May 7th).

### Plasmids construction

The expression vector pRK-HA, pCAGGS, pPB-EF1α-EGFP-puro, FMDV proteins recombinant plasmids fused with Flag-tag were preserved by Lanzhou Veterinary Research Institute. pCRISPR-sg4, *piggyBac* transposase expression vector pPBase and doxycycline-induced Cas9 expression vector pCRISPR-S10, preserved by the State Key Laboratory of Agricultural Biotechnology, China Agricultural University. The encoding region of TOB1 (Gene ID: 100144440), CEBPA (Gene ID: 397307), EGFR (Gene ID: 397070), and ERBB2 (Gene ID: 100516641) were PCR amplified using cDNA from IBRS-2 cells as a template. Then, TOB1 PCR products were cloned into the pPB-EF1α-EGFP-puro vector, CEBPA, EGFR and ERBB2 PCR products were cloned into the pRK-HA vector. All plasmids were confirmed by Sanger sequencing.

### Cell culture, viral infection, and transfection

PK-15, iPAM, HEK293T, and IBRS-2 cell lines were purchased from ATCC (USA). Cells were maintained in Dulbecco’s Modified Eagle Media (DMEM) supplementing with 10% fetal bovine serum (FBS), 100 U/mL penicillin, 100 μg/mL streptomycin and incubated at 37°C with 5% CO_2_. For transfection assays, IBRS2 cells were seeded into 6-well plates and transfected (approximately 80% confluent) with 2 μg plasmid DNA using JetPRIME (Polyplus) according to the manufacturer’s instructions. At 24 h post-transfection, cells were infected with FMDV O/BY/CHA/2010 at 37°C with 5% CO_2_. FMDV type O strain O/BY/CHA/2010 were obtained from the National Foot and Mouth Diseases Reference Laboratory, Lanzhou Veterinary Research Institute, Chinese Academy of Agricultural Sciences. At 1 h after infection, inoculum was removed, and replaced with 2 mL of fresh DMEM containing 10% FBS and 2% penicillin-streptomycin. After 12–24 h of infection, qRT-PCR assay were conducted. All experiments involved with FMDV were conducted in the Biosafety level 3 (BSL-3) facilities in Lanzhou Veterinary Research Institute of Chinese Academy of Agricultural Sciences approved by the Ministry of Agriculture and Rural Affairs.

### Construction of a genome-wide PB-CRISPR pig-KO library

Using Ensemble datasets of Sscrofa 10.2 version, we first designed 93,859 sgRNAs, that collectively targeted 16,886 protein-coding genes, 25 long ncRNAs, and 463 micro-RNAs. These sgRNAs were targeted to the exons near the 5’ end of transcripts. The number of off-targets in the genome and the type of mutations were used to rank these sgRNAs, and those with the lowest off-target scores were chosen. As a result, most genes have an average of 6 sgRNAs. In addition, we also designed a non-targeting sgRNA as a negative control. Then, we synthesized all these sgRNAs oligonucleotides and cloned into pCRISPR-sg4^23^ to obtain the PB-CRISPR-pig-KO library.

### Generation of mutant cell libraries and screening

To obtain the pig genome-wide mutant cell library, the PB-CRISPR library, pCRISPR-S10 and *piggyBac* transposase expression plasmids were co-transfected into IBRS-2 cells. Under the transient action of *piggyBac* transposase, the sgRNA expression element and puromycin resistance gene in PB-CRISPR library and the doxycycline-induced Cas9 expression element and neo gene in pCRISPR-S10 were integrated into the genome of IBRS-2 cells. For the CRISPR screening, ~1 × 10^8^ mutant cells were infected with FMDV at an MOI of 0.2 in DMEM without FBS and incubated at 37°C and 5% CO_2_. After 1.5 h incubation, the inoculum was removed and replaced with fresh DMEM supplementing with 2% FBS and 1% penicillin-streptomycin. At the 10th day after post-infection, viable cells were collected and expanded for the deep sequencing analysis and the next round of infection.

### Calculation of candidate gene ranking

As the sequences of sgRNA correspond to specific genes, theoretically, the enrichment of certain sgRNA in the surviving cell library after FMDV infection indicates that knockout of the corresponding genes enables cells to resist viral infection. In order to obtain a list of FMDV infection related genes, we remove the sequences on both sides of the sgRNA on the vector obtained from the second generation sequencing data, and then compare the obtained sgRNA sequences with the sequences in the PB-CRISPR library to determine the corresponding gene information of the sgRNA. Rank the results of NGS in each group according to the number of reads of sgRNA, from high to low. When the number of reads of a certain sgRNA/the number of reads of all sgRNAs in each group of sequencing results is > 0.0005%, then the sgRNA is valid data. Each effective sgRNA in each group of sequencing results has an Sg value, Sg = log_10_ (10000/rank). The score TS of each gene is (Sg1+Sg2+Sg3)^n^, where ‘n’ represents the number of Sg possessed by the gene, then rank all genes with TS values from high to low. Besides, we also compared the sgRNA frequencies in the mutant cell library before and after FMDV infection using MaGeCK software, and sorted them from high to low. Based on the above two rankings, the candidate gene ranking has been finally determined.

### Knockout of TOB1 in IBRS-2, PK-15 and iPAM cells by CRISPR/Cas9

Individual sgRNA targeting TOB1 gene or non-targeting sgRNA was cloned into the pCRISPR-sg4 vector. The pCRISPR-sg4 vector, pCRISPR-S10, and pPBase plasmids were co-transfected into IBRS-2, PK-15, and iPAM cells for 24 h, respectively. Cells were screened using culture media containing puromycin or G418. Then, cells were treated with culture media containing doxycycline for 5 days to induce Cas9 expression. Mixed knockout cells were seeded into 96-well plates. At the seventh day after transduction, the genotypes of cell colonies were analyzed by extracting genomic DNA and sequencing. The wild-type cells transfected with non-targeting sgRNA were as control group.

### RNA sequencing analysis

IBRS-2 control and TOB1-knockout IBRS-2 cells were harvested for RNA extraction using Trizol reagent (Invitrogen). RNA integrity was assessed using the Agilent 2100 RNA Nano 6000 Assay Kit (Agilent Technologies, CA, USA). Then the libraries were constructed using TruSeq PE Cluster Kit v3-cBot-HS (Illumina) according to the manufacturer’s instructions. The transcriptome sequencing and analysis were conducted by Trimmomatic and HISAT2 software. Differential expression analysis was performed using the DESeq FDR < 0.05 and fold change > 2 or fold change < 0.5 was set as the threshold for significantly differential expression gene (DEGs).

### Virus binding and internalization assays

For cell binding assays, IBRS-2 or PK-15 cells seeded on glass bottom cell culture dishes were incubated for 1 h at 4°C with equivalent amounts of FMDV particles. After extensive washes with cold PBS, the cells were fixed for 3 min with methanol at -20°C and analyzed by immunofluorescence with a rabbit antibody against the VP1 viral protein followed by Alexalabeled secondary antibodies. The surface of cells stained with MemBrite Fix Cell Surface Staining Kit (Biotium, 30093-T) and nuclei were labeled with DAPI Staining Solution (Beyotime, C1006). The cells were collected and quantified by qPCR. To quantify virus uptake, IBRS-2 or PK-15 cells seeded on glass bottom cell culture dishes were incubated with equal amounts of FMDV virus particles for 30 min at 37°C. Then, the cells were incubated with trypsin-EDTA for 5 min at 37°C to remove noninternalized particles and fixed with 4% PFA for 15 min at room temperature. After permeabilization with 0.1% Triton-100 for 5 min, the internalized particles were immunolabeled with rabbit anti-VP1 antibody followed by an Alexa Fluor 488-conjugated anti-rabbit antibody. The cells were collected and quantified by qPCR. Another group of cells were infected under the same conditions and RNA was extracted for subsequent qPCR detection.

### ELISA analysis

IFN-β level in cultured cell supernatants was evaluated using the Porcine IFN-β ELISA kit (SEKP-0046, Solarbio) according to the manufacturer’s instructions.

### Transfection of siRNAs

siRNAs were synthesized by Sangon Biotech (Shanghai, China). IBRS-2 and PK-15 cells were seeded into 12-well plates and transfected (approximately 60% confluent) with 80 pmol of siRNA using JetPRIME (Polyplus) according to the manufacturer’s instructions. A negative control siRNA (siRNA-NC) was also transfected into cells. At 24–48 h after transfection, the cells were used for further functional analysis.

### Real-time reverse transcription PCR (qRT-PCR) analysis

Total RNA from cells was extracted with Trizol Reagent (Invitrogen), then was measured using a NanoDrop 2000 spectrophotometer (Thermo Scientific) for assessing RNA quantity and quality. cDNAs were synthesized using the PrimeScrip RT reagent Kit (Takara). The qPCR reactions were prepared with RealUniversal SYBR Green Premix following the manufacturer’s instructions. Briefly, PCR mixtures (14 μL) contained 7 μL RealUniversal Premix, 0.6 μL forward primer (0.3 μM), 0.6 μL reverse primer (0.3 μM), and 5 μL cDNA template. The results were monitored using a CFX384 Real-Time PCR Detection System (Bio-Rad, USA) programmed for one cycle of 15 min at 95°C, followed by 39 cycles of 10 s at 95°C, 30 s at 60°C. The cDNA generated from the same amount of total RNA was used as a template to detect the expression of FMDV RNA and host cellular mRNA. The relative expression of mRNA was calculated based on the comparative cycle threshold (CT) (2^−ΔΔCT^) method. The glyceraldehyde-3-phosphate dehydrogenase (GAPDH) gene was used as a normalization control. For absolute quantitative real-time PCR, about 2 μL of viral RNAs were used as template to synthesize cDNAs. PCR mixtures contained 12.5 μL 2 × One Step RT-PCR buffer III, 0.5 μL Takara Ex Taq HS, 0.5 μL forward primer (10 μM), 0.5 μL reverse primer (10 μM), 1 μL 3D probe (10 μM) and add ddH_2_O to 25 μL. The results were monitored using a LightCycler 96 System (Roche) programmed for one cycle of 15 min at 42°C, followed by 40 cycles of 10 s at 99°C, 10 s at 55°C, 30 s at 72°C. The FMDV cDNA sequence encoding 3D protein (accession number: AH012984.2) was cloned into pMD19-T vector and used as a standard for the quantification of FMDV copy numbers.

### Immunoblot analysis

Approximately 2 × 10^6^ of IBRS-2 or PK-15 cells were lysed in cell lysis buffer (50 mM Tris-HCl [pH = 6.8], 2% SDS, 10% glycerol, 1% β-mercaptoethanol, 12.5 mM EDTA, 0.02% bromophenol blue) supplemented with protease inhibitor and phenylmethylsulfonyl fluoride (PMSF), with cell lysates precleared at 4°C by centrifugation at 12,000 rpm for 10 min. The precleared lysates were separated by 10% polyacrylamide gel SDS. Separated proteins were then transferred onto a nitrocellulose membrane and probed with TOB1 (Proteintech, #14915-1-AP, 1:1000), with β-actin (Proteintech, #66009-lg, 1:5000) used as an internal loading control. The primary antibodies were detected with horseradish peroxidase (HRP) conjugated goat anti-rabbit IgG or goat anti-mouse IgG, and the secondary antibodies were visualized by ECL Prime Western Blotting Detection Reagents (TIANGEN, #X0926).

### Co-immunoprecipitation assay

Co-immunoprecipitation was performed to confirm protein interactions. HEK293T cells were seeded in 10-cm dishes and co-transfected with different doses of plasmids for 24 h. The samples were collected, washed with phosphate-buffered saline (PBS), and lysed by NP40 lysis buffer. The lysates were ultrasonicated, freezed, and thawed. Following centrifugation, the lysates were incubated with protein G beads and primary antibodies at 4°C, with gentle shaking. The samples were washed with PBS for three times and dissociated in 2 × SDS loading buffer with 100°C boiling. The precipitates were analyzed by immunoblot assay as described above.

### Establishment of TOB1 knockout mice using the CRISPR/Cas9 system

TOB1^-/-^ and TOB1^+/+^ mice on the C57BL/6J background were generated at the Shanghai Model Organisms Center, Inc. (Shanghai, China), using CRISPR/Cas9 technology to repair nonhomologous recombination to introduce mutations resulted in frame-shifting of the TOB1 protein and loss of function. PCR products were sequenced to analyze the frame-shifting of the target gene protein in F0 mice. Positive F0 generation mice were selected to mate with wild-type C57BL/6J mice to obtain F1 generation heterozygous mice. Genotyping by PCR was performed using a combination of the following primers: 1, 5’ -TCCTGCTGGGAAGTGGTATG-3’; 2, 5’-ACCAAGCCTGAATGTGTCCTT-3’; 3, 5’-CCAGCGGTCATTTCCAGTCT-3’; and 4, 5’-TCCACGTACAGCACCTTCAC-3’. Amplification of the wild-type allele with primers 1 and 2 gave rise to a 787-bp fragment, while amplification of the mutant allele primers 3 and 4 led to no fragment. Western blot confirmed that TOB1 was completely knocked out in the established mice. TOB1^-/-^ mice were bred under specific-pathogen-free conditions. Three-day-old male and female mice were randomly allocated for each experimental group, and littermates were used as controls.

### Virus infection in suckling mice

These TOB1^-/-^ mice were born at a standard Mendelian ratio and did not show any developmental or behavioral abnormality compared with their wild-type littermates. Three-day-old TOB1^-/-^ and TOB1^+/+^ (n = 6 in each group) suckling mice were used and divided into three groups. One group was subcutaneously injected with equal amounts of PBS, and the other two groups were injected subcutaneously with two concentrations of FMDV (7 × 10^3^ PFU and 7 × 10^4^ PFU). The survival rate was monitored every 24 h.

### Statistical analysis

Statistical analysis was performed using R programming language. The means ± S.D. was determined for each treatment group in the separated experiments. Two-tailed Student’s *t-*test was used to determine significant differences between treatment and control groups (*P < 0.05; **P < 0.01; ***P < 0.001; ns, no significant).

## Supporting information

S1 Fig Screening of FMDV resistance genes and construction of candidate genes knockout monoclonal cell lines. **a** The expression and protein level of Cas9 in mutant cell libraries and control library with or without doxorubicin-induced. **b** The process of mutant cell library construction including plasmid transfection, screening of transfected cells, and doxorubicin induced Cas9 expression. **c** The mutant cell libraries and control library were infected with FMDV of 0.1 MOI for 36 h. After 10 days, these FMDV-resistant cells were enriched in the mutant cell Library 1, Library 2, and Library 3. **d** Alignment of the nucleic acid sequences of monoclonal knockout cells of thirty candidate genes with control cells. Control library, transfect the PB-CRISPR plasmid library inserted with non-targeting sgRNA.
(TIF)

S2 Fig Phenotypic validation of TOB1-knockout cells with FMDV infection. **a** Wild-type, IBRS-2 control, and TOB1-knockout IBRS-2 cells were infected with FMDV of 0.1 MOI for 12 h. The morphologic change of cells was observed under inverted microscope. **b** Wild-type, IBRS-2 control, and TOB1-knockout IBRS-2 cells were infected with FMDV of 0.1 MOI at indicated time points. The FMDV RNA copies were measured by absolute quantitative real-time PCR. **c** IBRS-2 control and TOB1-knockout IBRS-2 cells were infected with FMDV of 0.1 MOI for 4, 8, and 12 h. The samples were subjected to immunofluorescence using anti-VP3 antibody. **d** The cell viability of wild-type, IBRS-2 control, and TOB1-knockout IBRS-2 cells, infected with FMDV of 0.1 MOI for 12, 16, 20, and 24 h. **e** Absolute quantitative real-time PCR for determination of FMDV RNA copies number in TOB1-knockout iPAM cells with FMDV infection at 0.1 MOI for 6, 12, and 24 h. Data shown includes technical replicates from a single experiment and is representative of three independent experiments (e). Data are represented as means ± S.D.; *P < 0.05; **P < 0.01; ***P < 0.001; ns, no significant. *P* values were determined by two-sided Student’s *t*-test.
(TIF)

S3 Fig Detection of IFN pathway in TOB1-knockout cells. **a** The clustering analysis of DEGs between the three IBRS-2 control cells samples (WT1, WT2, and WT3) and the three TOB1-knockout IBRS-2 cells samples (KO1, KO2, and KO3). Using the FPKM values of differentially expressed genes in the WT and KO groups as an index of expression levels, hierarchical clustering analysis was performed to compare expression differences. Different colored regions represent different clustering information (red: upregulation, green: downregulation). **b** The transcriptional levels of ISGs in TOB1-knockout iPAM cells were measured by qPCR. **c** HEK293T cells were transfected with IFN-β-luc, ISRE-luc, or IRF1-luc and hTOB1-Flag or pTOB1-Flag plasmid (100 ng) for 24 h. Cells were stimulated with IFN-β or IFN-γ for another 12 h, and whole-cell lysates were collected for measurements of luciferase activity. **d** The protein level of IFN-β in control and TOB1-knockout IBRS-2, PK-15, and iPAM cells were detected by ELISA. **e** IBRS-2 control and TOB1-knockout IBRS-2 cells were treated with IFN-β for 0, 4, 8, and 12 h. The expression of *JAK1*, *TYK2*, *STAT1*, *STAT2*, *IRF9*, and *STAT3* were measured by qPCR. Data shown includes technical replicates from a single experiment and is representative of three independent experiments (b, c, d, e). Data are represented as means ± S.D.; *P < 0.05; **P < 0.01; ***P < 0.001; ns, no significant. *P* values were determined by two-sided Student’s *t*-test.
(TIF)

S4 Fig The role of CEBPA in RIG-I mediated antiviral innate immunity. **a** IBRS-2 cells were transfected with three siRNAs for 24 h. The interference efficiency of *CEBPA* was detected by qPCR. **b** TOB1-knockout IBRS-2 cells were transfected with siRNA for 24 h, and then infected with FMDV of 0.1 MOI for 24 h. The FMDV RNA copies number were detected by absolute quantitative real-time PCR. **c** IBRS-2 cells were transfected with plasmid encoding CEBPA for 24 h. The expression of *IFNA*, *IFNB*, *ISG15*, and *ISG56* were measured by qPCR. **d** IBRS-2 cells were transfected with three siRNAs for 24 h. The interference efficiency of *RIG-I* was detected by qPCR. **e** IBRS-2 cells were transfected with RIG-I siRNA for 24 h. The expression of *IFNB*, *ISG15*, and *MX1* were measured by qPCR. **f** IBRS-2 control and TOB1-knockout IBRS-2 cells were transfected with plasmids encoding ISG15 and MX1 for 24 h, and then infected with FMDV of 0.1 MOI for 24 h. The FMDV RNA copies number were detected by absolute quantitative real-time PCR. Data shown includes technical replicates from a single experiment and is representative of three independent experiments (a, b, c, d, e, f). Data are represented as means ± S.D.; *P < 0.05; **P < 0.01; ***P < 0.001; ns, no significant. *P* values were determined by two-sided Student’s *t*-test.
(TIF)

S5 Fig The effect of EGFR/ERBB2 pathway on FMDV infection. **a, b** iPAM control and TOB1-knockout iPAM cells were infected with FMDV (MOI of 0.1, 1 and 10) for 1 h at 4°C (a) or 30 min at 37°C (b). The samples were subjected to immunofluorescence using anti-VP3 antibody. The replication levels of FMDV were quantified by qPCR.**c** iPAM control cells were treated with EGFR/ERBB2 tyrosine kinase domain inhibitor (Lapatinib) or DMSO at 0.03, 0.3, 3, 30, 300 μM for 24 h. Then, cells were infected with FMDV for 12 h at 0.01 MOI. The FMDV RNA copies number were detected by absolute quantitative real-time PCR. PC, positive control. Mock, uninfected group. L, Lapatinib. D, DMSO. **d** IBRS-2 control and TOB1-knockout IBRS-2 cells were treated with Erlotinib, Gefitinib at 10 μM for 24 h, or EGF 10 μM for 10 min. The replication levels of FMDV were detected by qPCR. **e** The cell viability of wild-type IBRS-2 and PK-15 cells, treated with Erlotinib or Gefitinib at 0, 2, 5, 10, 50, 100 μM for 24 h. **f** Wild-type IBRS-2 cells were transfected with three siRNAs for 24 h. The interference efficiency of *EGFR* and *ERBB2* was detected by qPCR and Western blot. **g** IBRS-2 and PK-15 cells were transfected with EGFR or ERBB2 siRNAs for 24 h, and then infected with FMDV (MOI of 10) for 30 min at 37°C. The FMDV RNA copies number were detected by absolute quantitative real-time PCR. **h** EGFR or ERBB2-knockout IBRS-2 cells were infected with FMDV for 12 h at 0.01 MOI. The replication levels of FMDV were detected by qPCR. **i** TOB1-knockout IBRS-2 cells were transfected with pRK-EGFR-HA plasmid for 24 h, and then infected with FMDV for 12 h at 0.01 MOI. The FMDV RNA copies number were detected by absolute quantitative real-time PCR. EV, empty vector. Data shown includes technical replicates from a single experiment and is representative of three independent experiments (a, b, c, d, e, f, g, h). Data are represented as means ± S.D.; *P < 0.05; **P < 0.01; ***P < 0.001; ns, no significant. *P* values were determined by two-sided Student’s *t*-test.
(TIF)

S1 Data The data of Western blot in Figs 1–7. (PDF)

S1 Table The values used to build graphs in Figs 1–7. (XLSX)
