# Supplementary figures and images for: A genome-wide CRISPR screening uncovers that TOB1 acts as a key host factor for FMDV infection via both IFN and EGFR mediated pathways
Source: PLoS Pathog. 2024 Mar 21;20(3):e1012104. doi: 10.1371/journal.ppat.1012104 (PMC10986976; doi:10.1371/journal.ppat.1012104)

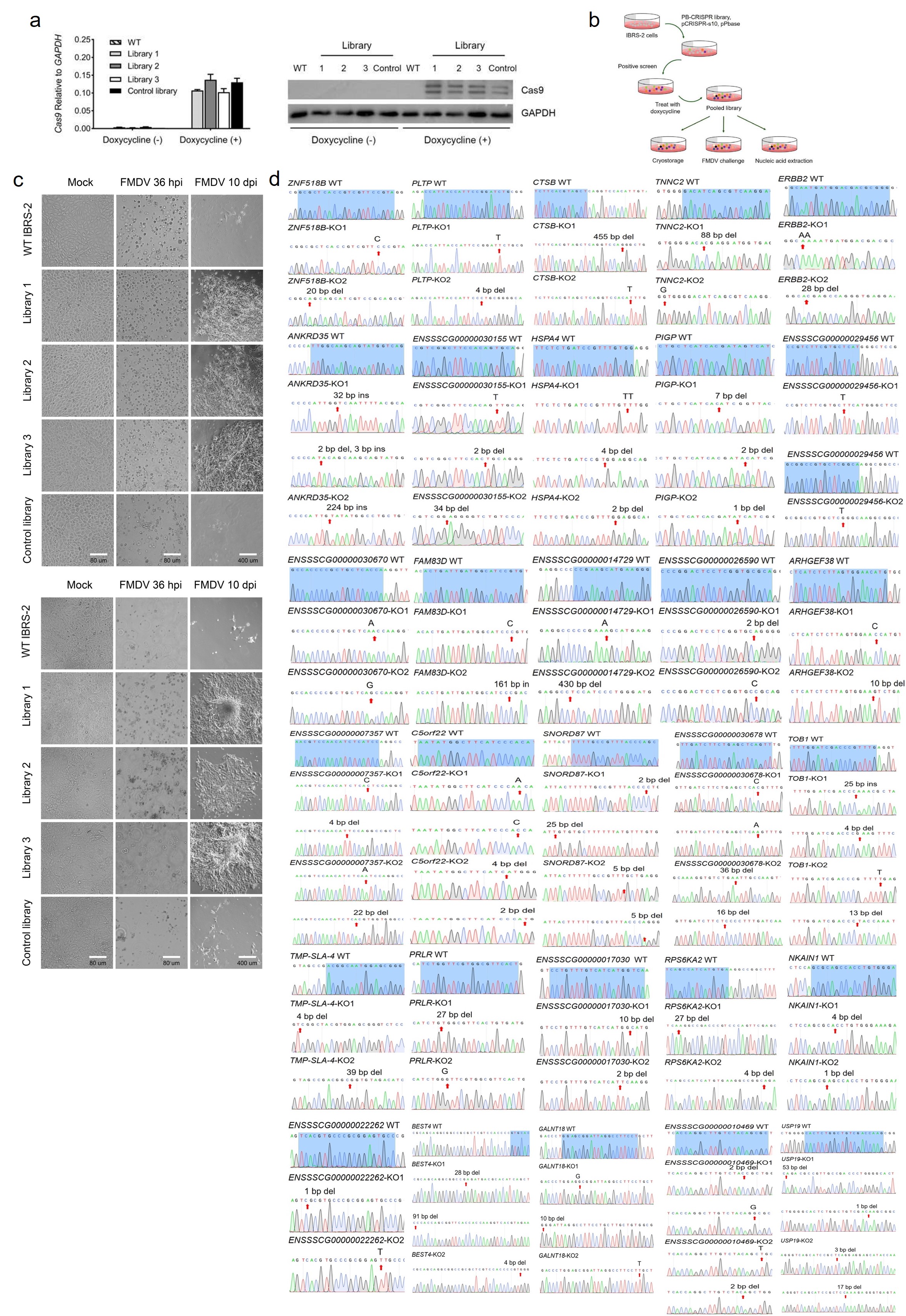

Supplement: S1 Fig — a The expression and protein level of Cas9 in mutant cell libraries and control library with or without doxorubicin-induced. b The process of mutant cell library construction including plasmid transfection, screening of transfected cells, and doxorubicin induced Cas9 expression. c The mutant cell libraries and control library were infected with FMDV of 0.1 MOI for 36 h. After 10 days, these FMDV-resistant cells were enriched in the mutant cell Library 1, Library 2, and Library 3. d Alignment of the nucleic acid sequences of monoclonal knockout cells of thirty candidate genes with control cells. Control library, transfect the PB-CRISPR plasmid library inserted with non-targeting sgRNA. (TIF) [file ppat.1012104.s001.tif]

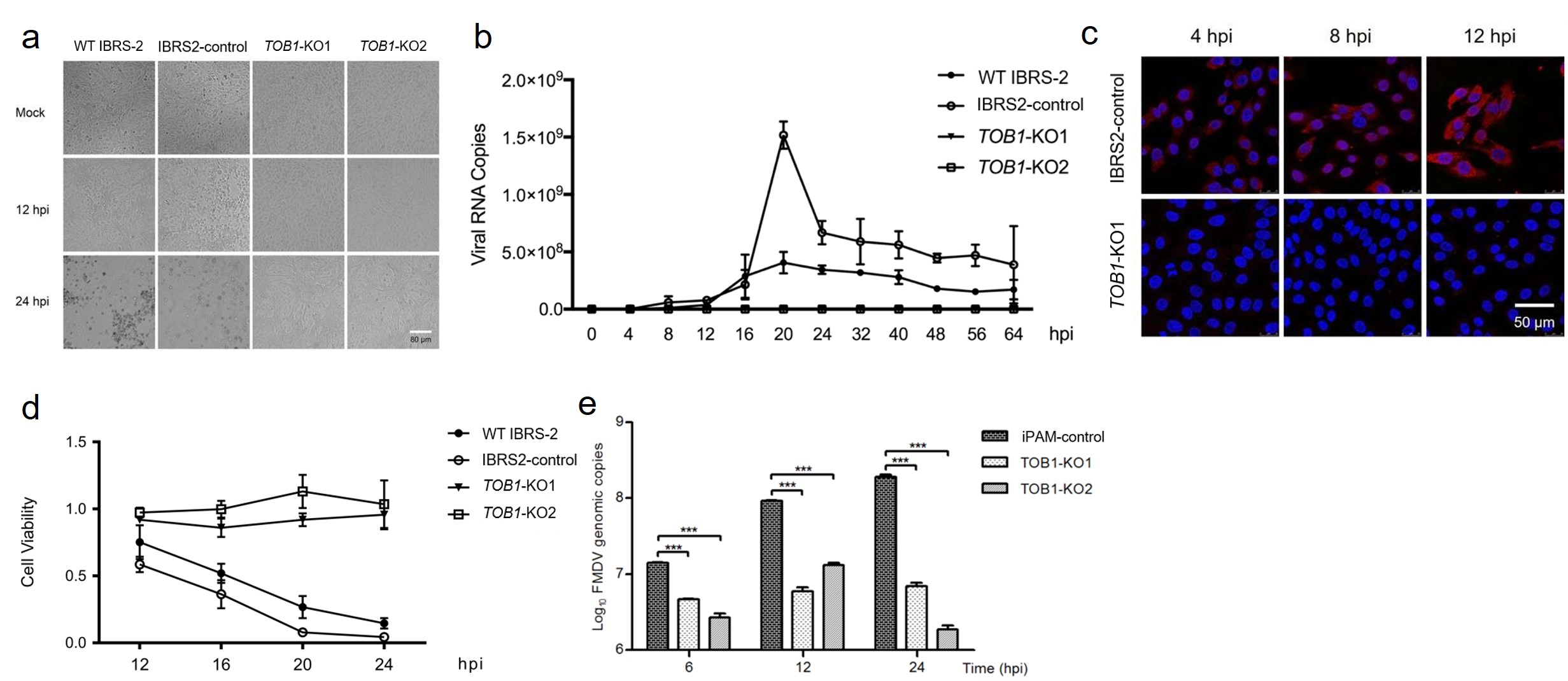

Supplement: S2 Fig — a Wild-type, IBRS-2 control, and TOB1-knockout IBRS-2 cells were infected with FMDV of 0.1 MOI for 12 h. The morphologic change of cells was observed under inverted microscope. b Wild-type, IBRS-2 control, and TOB1-knockout IBRS-2 cells were infected with FMDV of 0.1 MOI at indicated time points. The FMDV RNA copies were measured by absolute quantitative real-time PCR. c IBRS-2 control and TOB1-knockout IBRS-2 cells were infected with FMDV of 0.1 MOI for 4, 8, and 12 h. The samples were subjected to immunofluorescence using anti-VP3 antibody. d The cell viability of wild-type, IBRS-2 control, and TOB1-knockout IBRS-2 cells, infected with FMDV of 0.1 MOI for 12, 16, 20, and 24 h. e Absolute quantitative real-time PCR for determination of FMDV RNA copies number in TOB1-knockout iPAM cells with FMDV infection at 0.1 MOI for 6, 12, and 24 h. Data shown includes technical replicates from a single experiment and is representative of three independent experiments (e). Data are represented as means ± S.D.; *P < 0.05; **P < 0.01; ***P < 0.001; ns, no significant. P values were determined by two-sided Student’s t-test. (TIF) [file ppat.1012104.s002.tif]

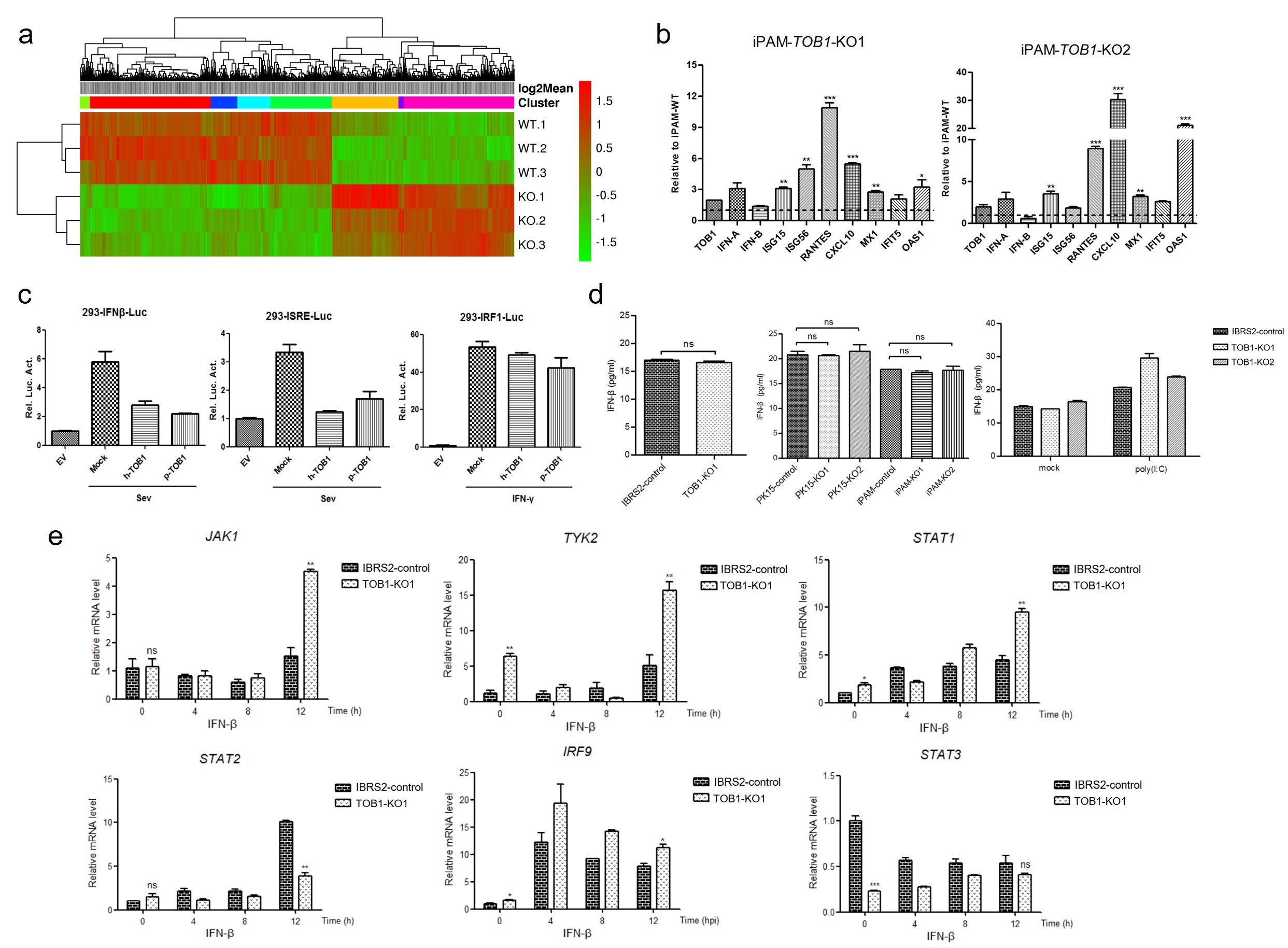

Supplement: S3 Fig — a The clustering analysis of DEGs between the three IBRS-2 control cells samples (WT1, WT2, and WT3) and the three TOB1-knockout IBRS-2 cells samples (KO1, KO2, and KO3). Using the FPKM values of differentially expressed genes in the WT and KO groups as an index of expression levels, hierarchical clustering analysis was performed to compare expression differences. Different colored regions represent different clustering information (red: upregulation, green: downregulation). b The transcriptional levels of ISGs in TOB1-knockout iPAM cells were measured by qPCR. c HEK293T cells were transfected with IFN-β-luc, ISRE-luc, or IRF1-luc and hTOB1-Flag or pTOB1-Flag plasmid (100 ng) for 24 h. Cells were stimulated with IFN-β or IFN-γ for another 12 h, and whole-cell lysates were collected for measurements of luciferase activity. d The protein level of IFN-β in control and TOB1-knockout IBRS-2, PK-15, and iPAM cells were detected by ELISA. e IBRS-2 control and TOB1-knockout IBRS-2 cells were treated with IFN-β for 0, 4, 8, and 12 h. The expression of JAK1, TYK2, STAT1, STAT2, IRF9, and STAT3 were measured by qPCR. Data shown includes technical replicates from a single experiment and is representative of three independent experiments (b, c, d, e). Data are represented as means ± S.D.; *P < 0.05; **P < 0.01; ***P < 0.001; ns, no significant. P values were determined by two-sided Student’s t-test. (TIF) [file ppat.1012104.s003.tif]

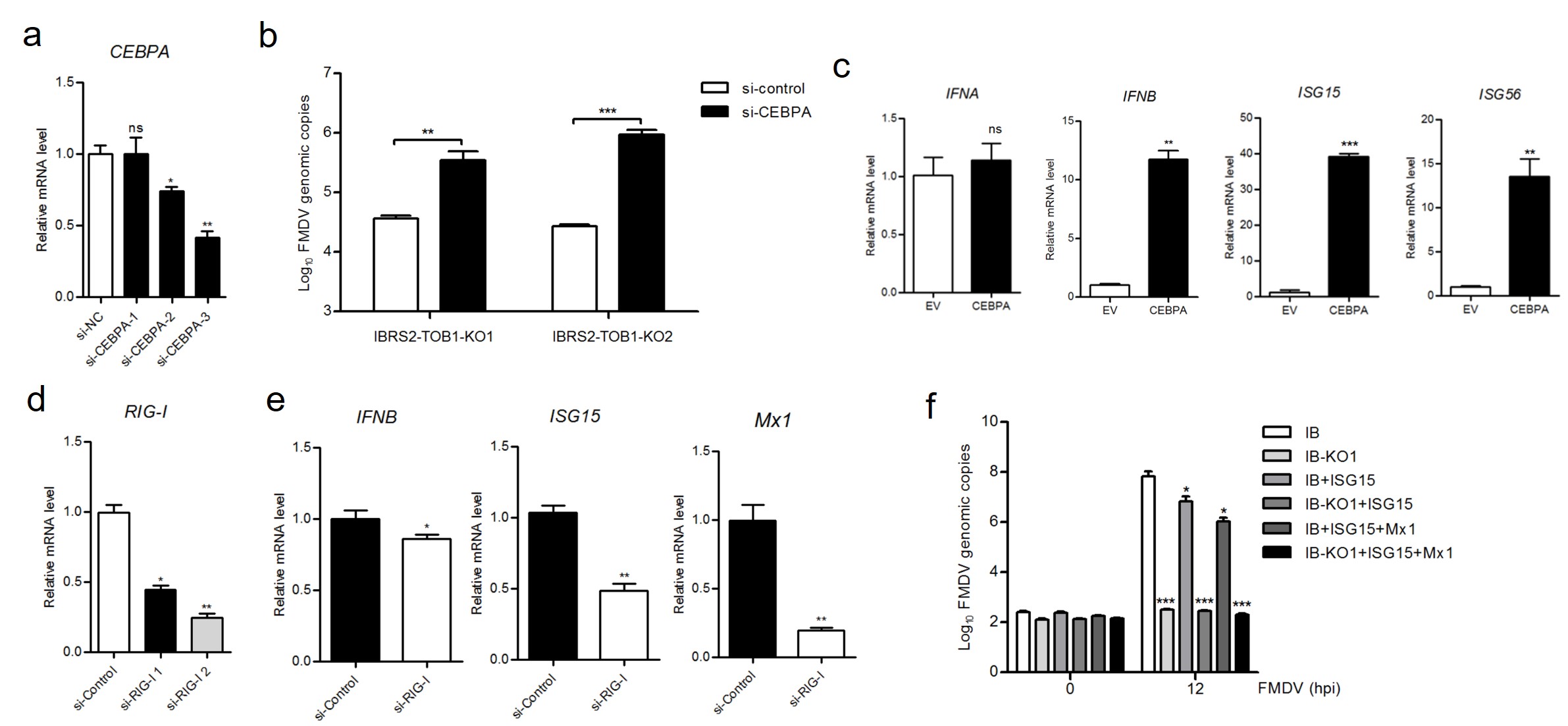

Supplement: S4 Fig — a IBRS-2 cells were transfected with three siRNAs for 24 h. The interference efficiency of CEBPA was detected by qPCR. b TOB1-knockout IBRS-2 cells were transfected with siRNA for 24 h, and then infected with FMDV of 0.1 MOI for 24 h. The FMDV RNA copies number were detected by absolute quantitative real-time PCR. c IBRS-2 cells were transfected with plasmid encoding CEBPA for 24 h. The expression of IFNA, IFNB, ISG15, and ISG56 were measured by qPCR. d IBRS-2 cells were transfected with three siRNAs for 24 h. The interference efficiency of RIG-I was detected by qPCR. e IBRS-2 cells were transfected with RIG-I siRNA for 24 h. The expression of IFNB, ISG15, and MX1 were measured by qPCR. f IBRS-2 control and TOB1-knockout IBRS-2 cells were transfected with plasmids encoding ISG15 and MX1 for 24 h, and then infected with FMDV of 0.1 MOI for 24 h. The FMDV RNA copies number were detected by absolute quantitative real-time PCR. Data shown includes technical replicates from a single experiment and is representative of three independent experiments (a, b, c, d, e, f). Data are represented as means ± S.D.; *P < 0.05; **P < 0.01; ***P < 0.001; ns, no significant. P values were determined by two-sided Student’s t-test. (TIF) [file ppat.1012104.s004.tif]

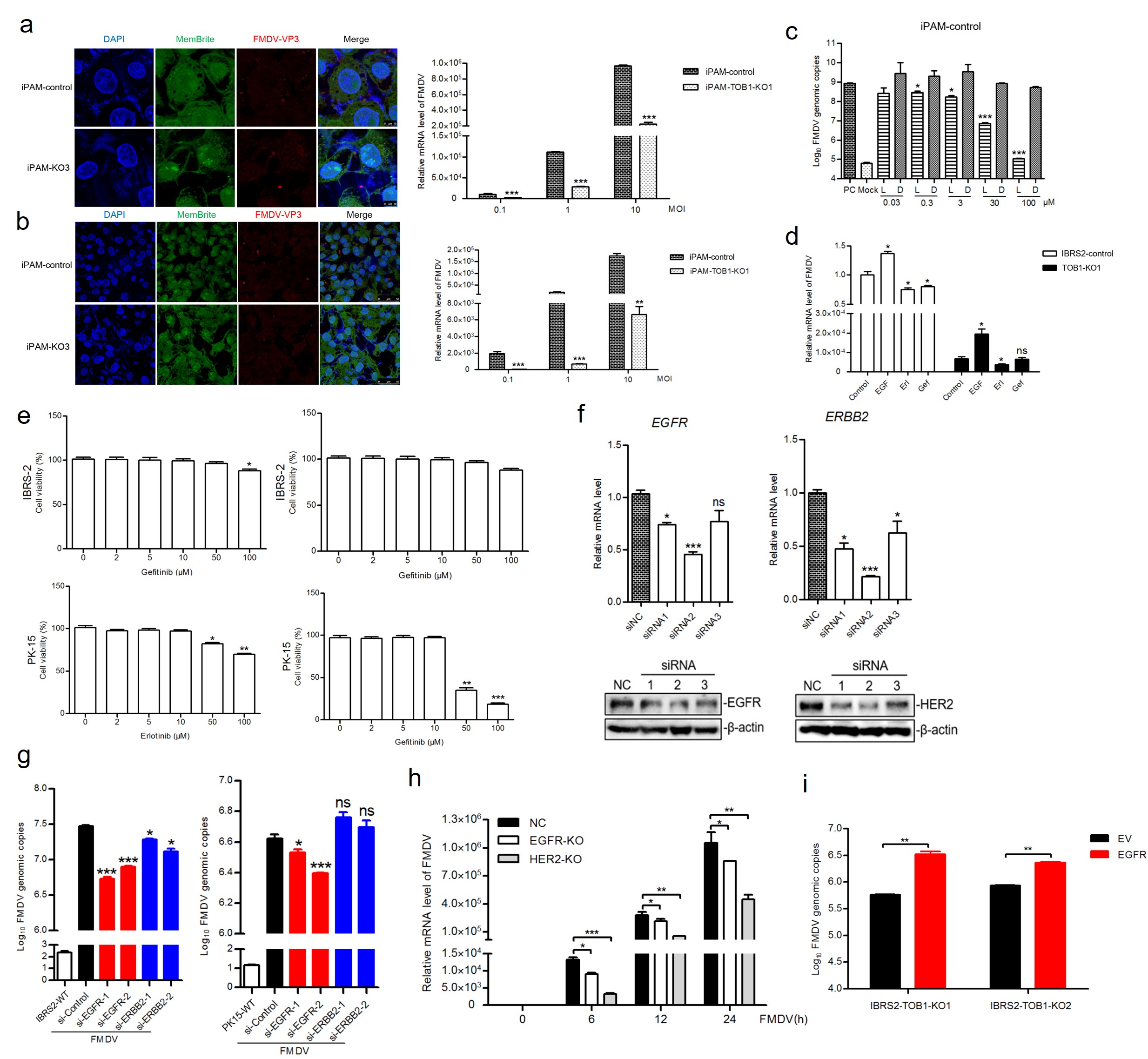

Supplement: S5 Fig — a, b iPAM control and TOB1-knockout iPAM cells were infected with FMDV (MOI of 0.1, 1 and 10) for 1 h at 4°C (a) or 30 min at 37°C (b). The samples were subjected to immunofluorescence using anti-VP3 antibody. The replication levels of FMDV were quantified by qPCR.c iPAM control cells were treated with EGFR/ERBB2 tyrosine kinase domain inhibitor (Lapatinib) or DMSO at 0.03, 0.3, 3, 30, 300 μM for 24 h. Then, cells were infected with FMDV for 12 h at 0.01 MOI. The FMDV RNA copies number were detected by absolute quantitative real-time PCR. PC, positive control. Mock, uninfected group. L, Lapatinib. D, DMSO. d IBRS-2 control and TOB1-knockout IBRS-2 cells were treated with Erlotinib, Gefitinib at 10 μM for 24 h, or EGF 10 μM for 10 min. The replication levels of FMDV were detected by qPCR. e The cell viability of wild-type IBRS-2 and PK-15 cells, treated with Erlotinib or Gefitinib at 0, 2, 5, 10, 50, 100 μM for 24 h. f Wild-type IBRS-2 cells were transfected with three siRNAs for 24 h. The interference efficiency of EGFR and ERBB2 was detected by qPCR and Western blot. g IBRS-2 and PK-15 cells were transfected with EGFR or ERBB2 siRNAs for 24 h, and then infected with FMDV (MOI of 10) for 30 min at 37°C. The FMDV RNA copies number were detected by absolute quantitative real-time PCR. h EGFR or ERBB2-knockout IBRS-2 cells were infected with FMDV for 12 h at 0.01 MOI. The replication levels of FMDV were detected by qPCR. i TOB1-knockout IBRS-2 cells were transfected with pRK-EGFR-HA plasmid for 24 h, and then infected with FMDV for 12 h at 0.01 MOI. The FMDV RNA copies number were detected by absolute quantitative real-time PCR. EV, empty vector. Data shown includes technical replicates from a single experiment and is representative of three independent experiments (a, b, c, d, e, f, g, h). Data are represented as means ± S.D.; *P < 0.05; **P < 0.01; ***P < 0.001; ns, no significant. P values were determined by two-sided Student’s t-test. (TIF) [file ppat.1012104.s005.tif]
