# Supplementary material for: A genome-wide CRISPR screening uncovers that TOB1 acts as a key host factor for FMDV infection via both IFN and EGFR mediated pathways
Source: PLoS Pathog. 2024 Mar 21;20(3):e1012104. doi: 10.1371/journal.ppat.1012104 (PMC10986976; doi:10.1371/journal.ppat.1012104)

Figure 2B

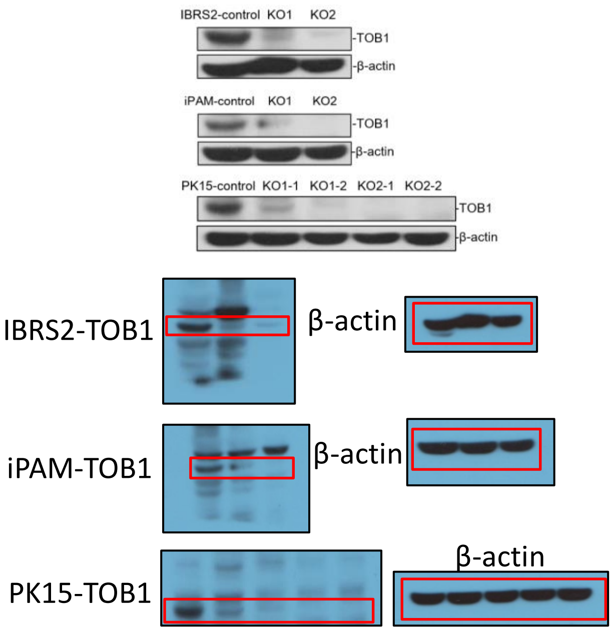

Figure 2E

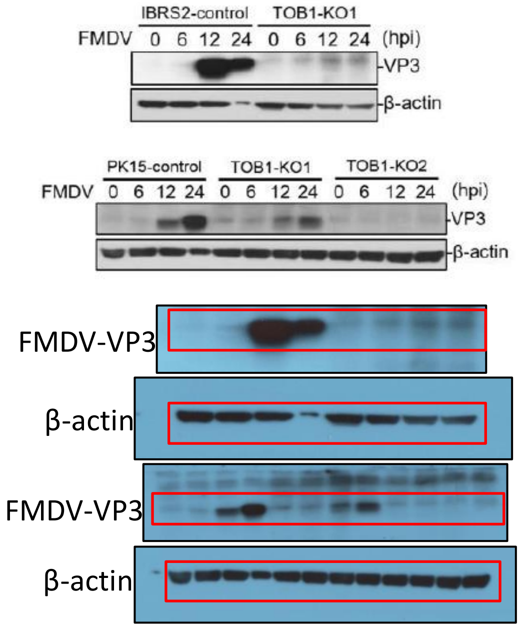

Figure 4E

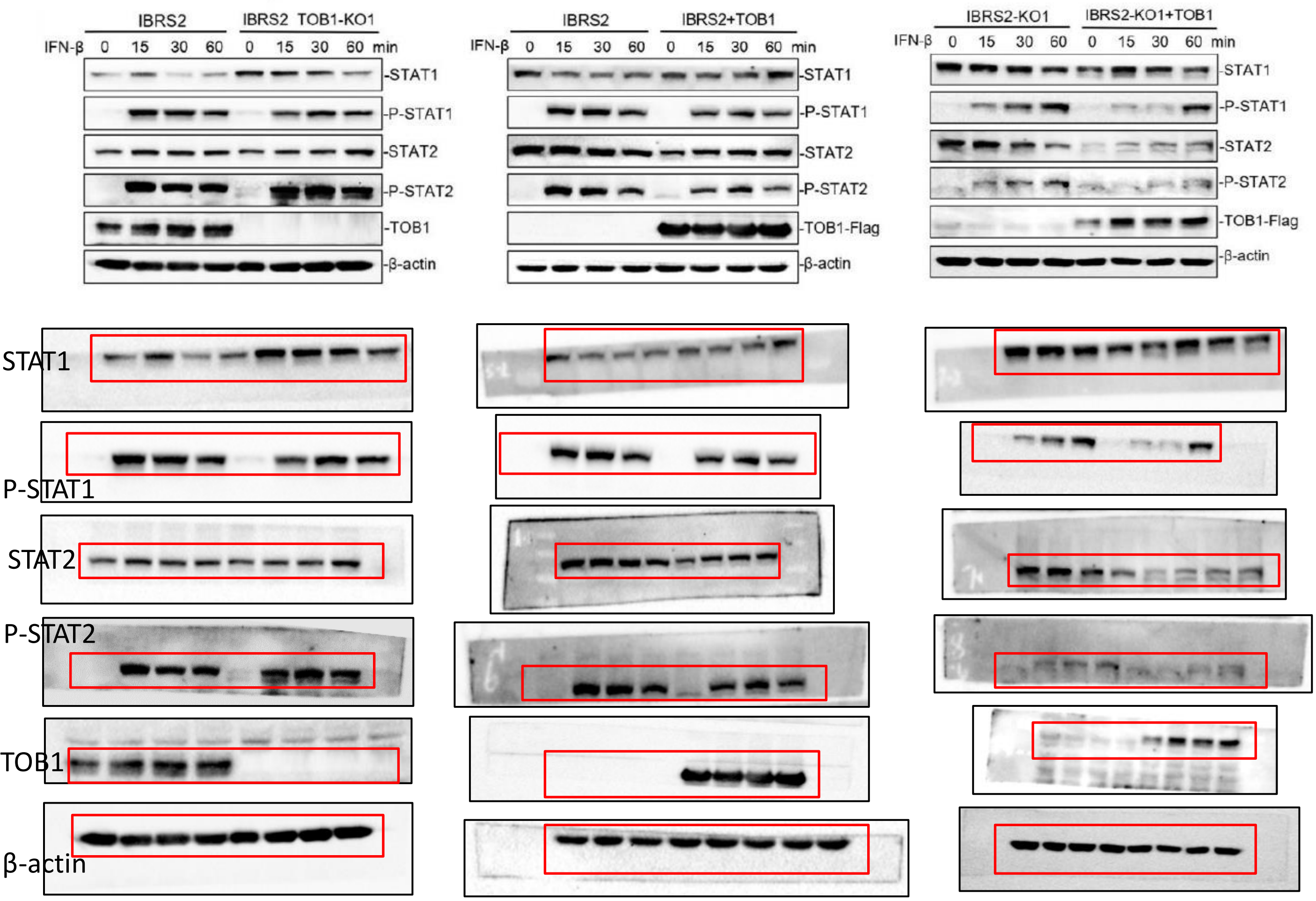

Figure 4F

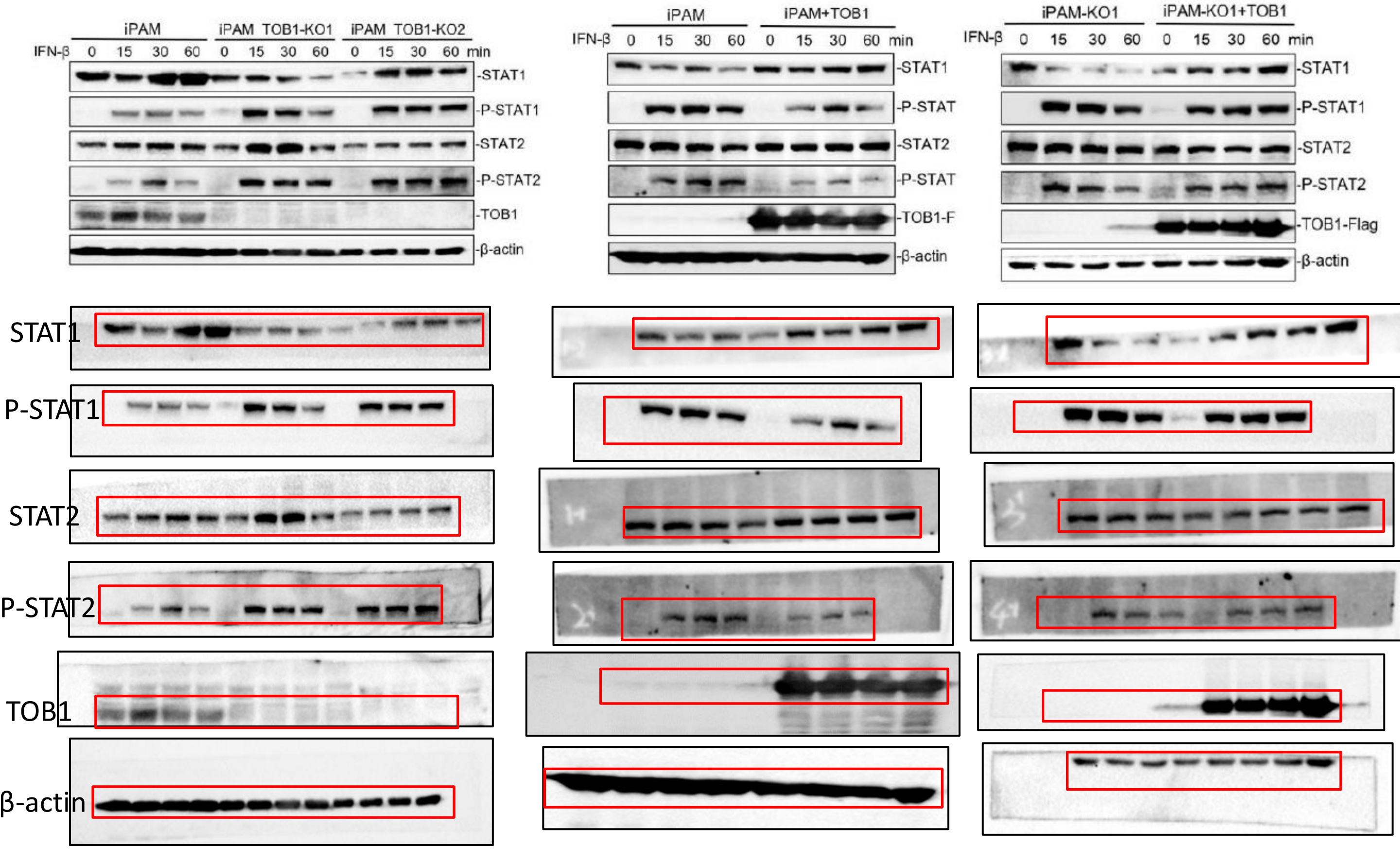

Figure 5B

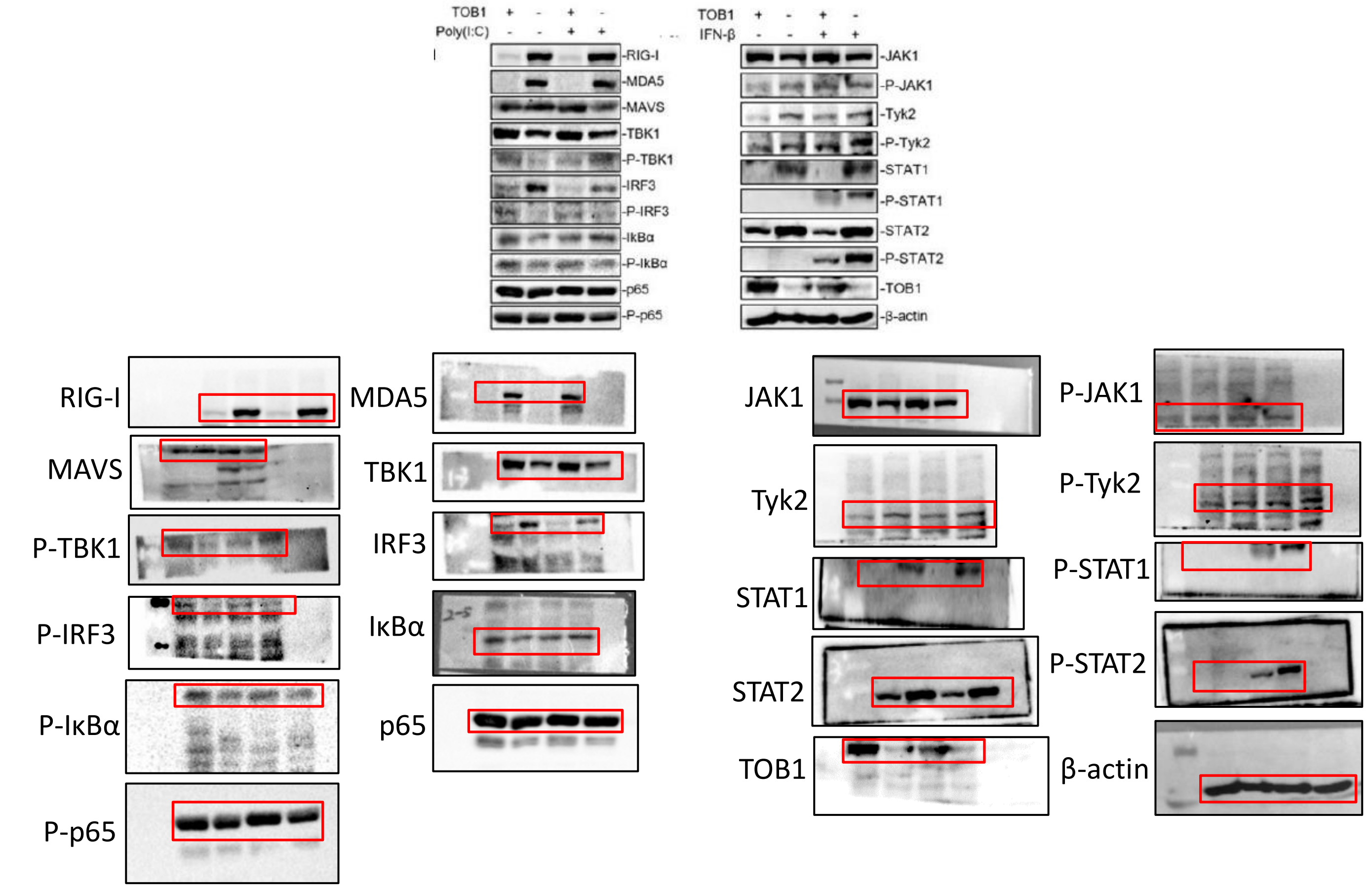

Figure 5C

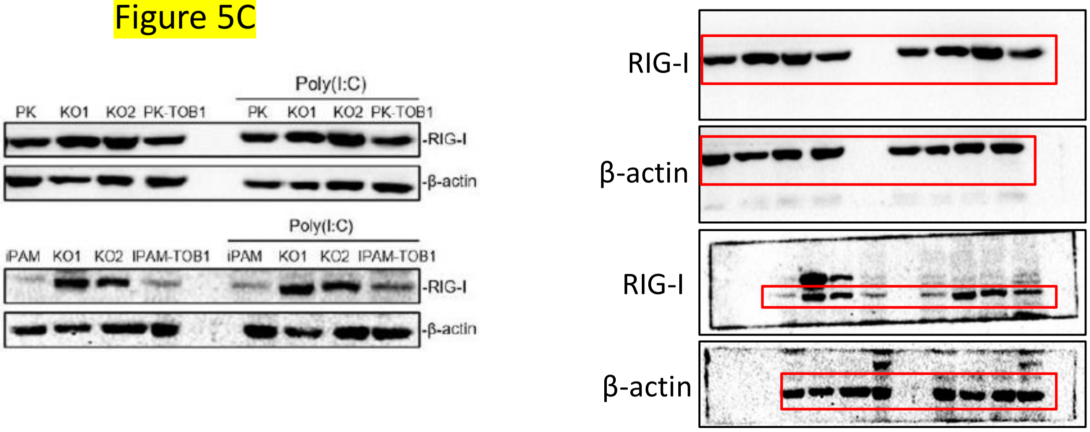

Figure 6E

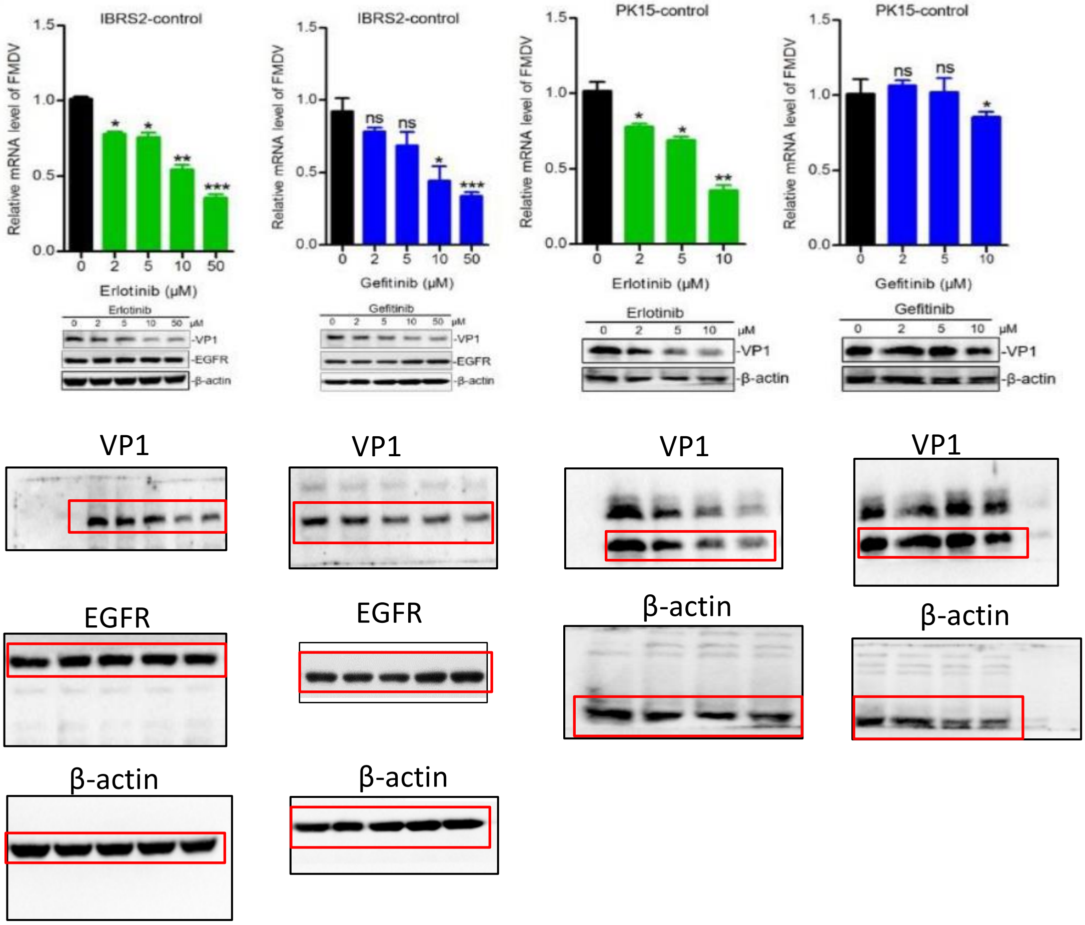

Figure 6H

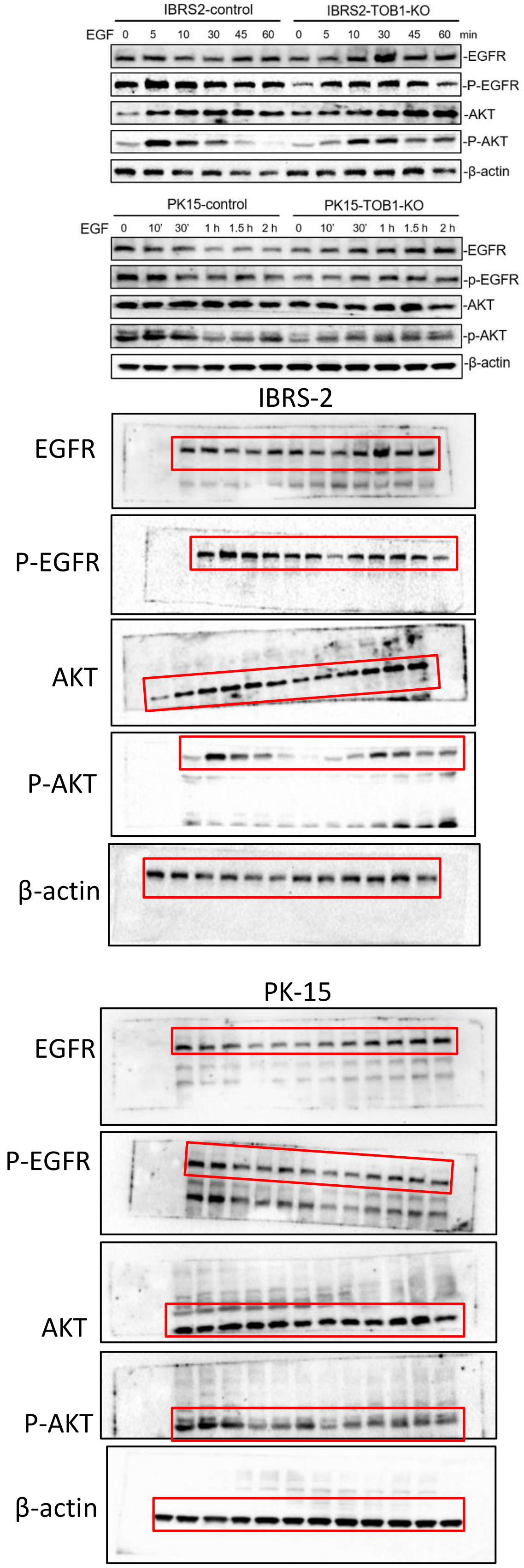

Figure 6I

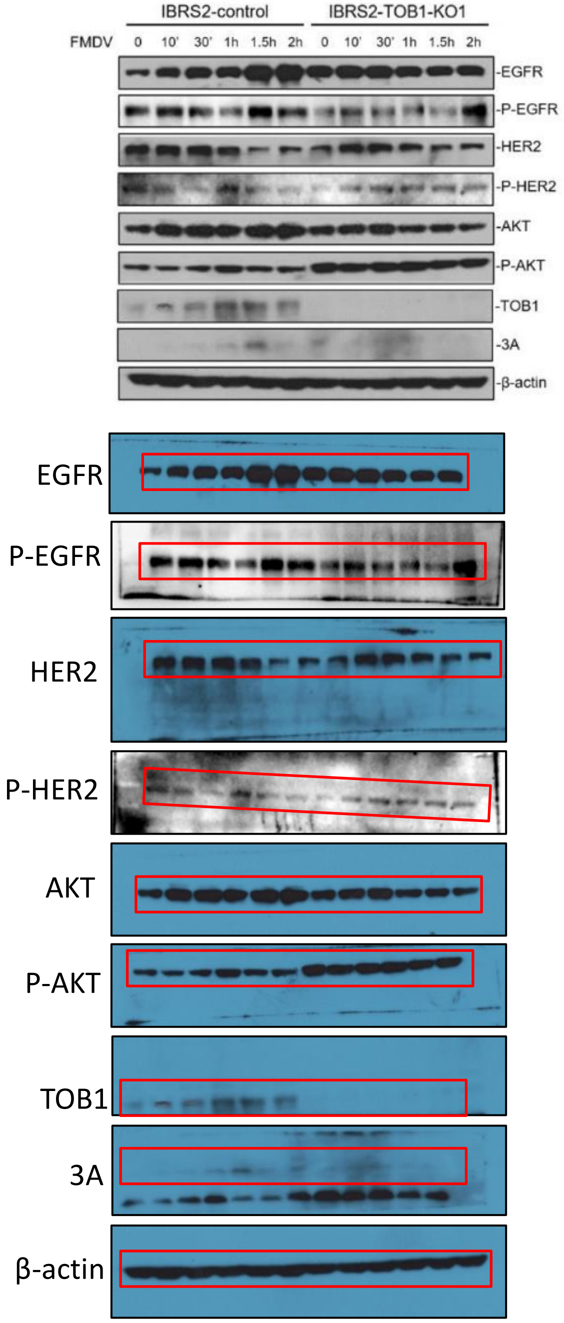

Figure 6J

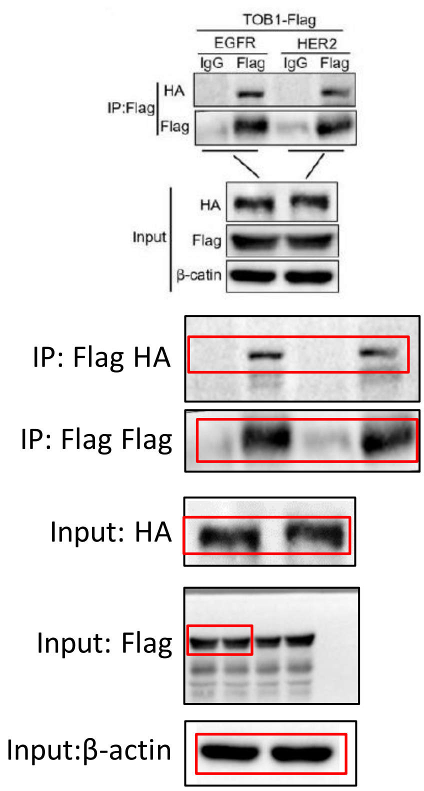

Figure 6K

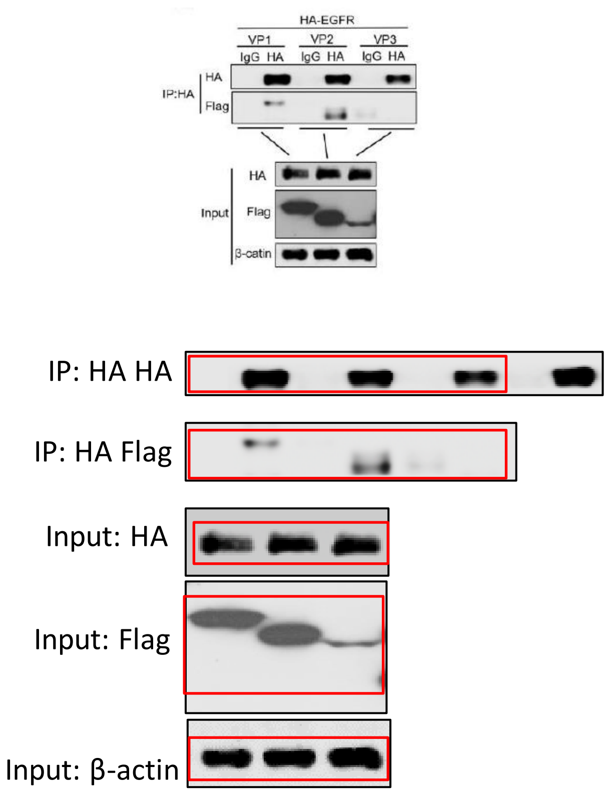

Figure 7H

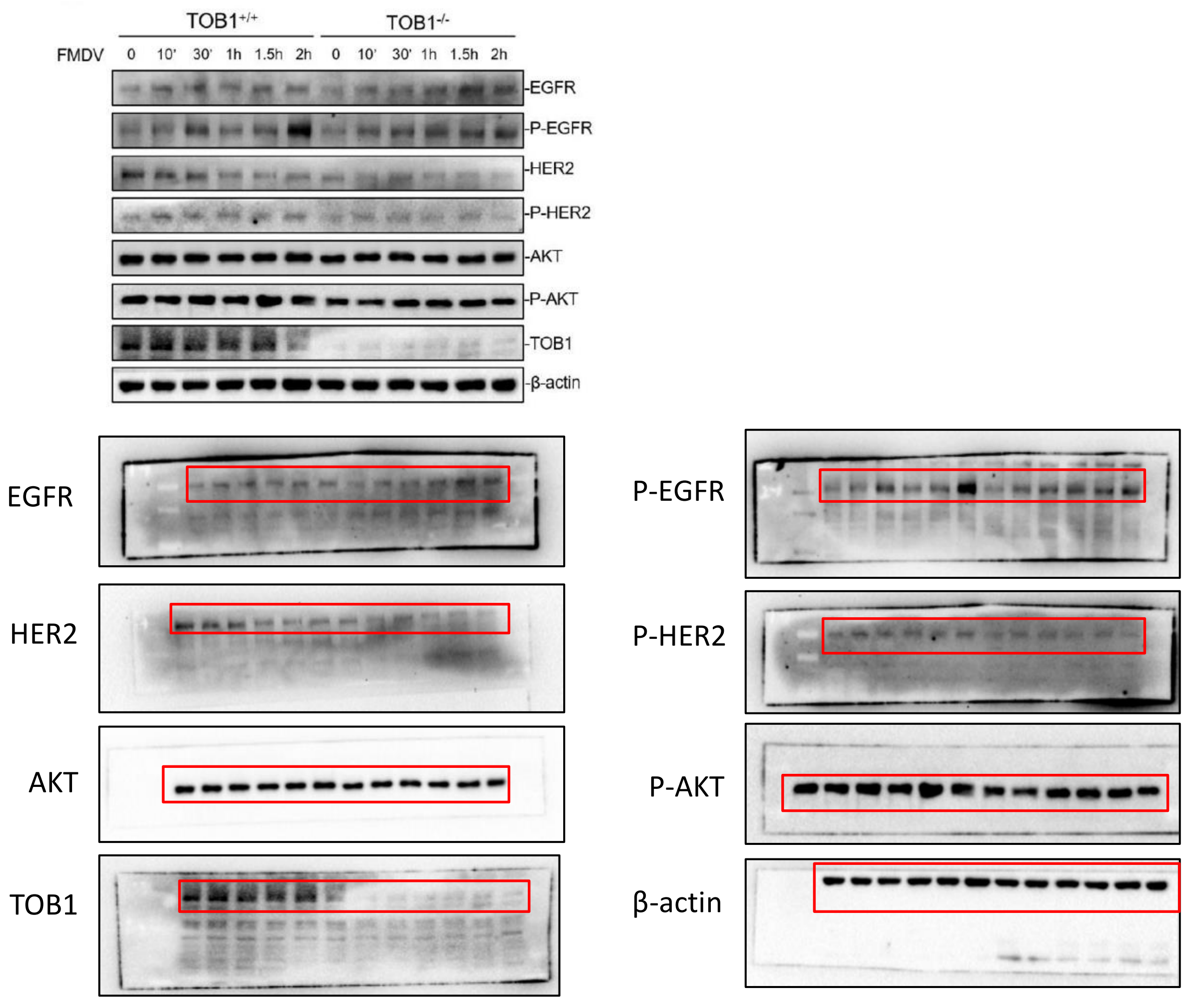

Supplement: S1 Data — (PDF) [file ppat.1012104.s006.pdf]
